# Characterizing the “fungal shunt”: Parasitic fungi on diatoms affect carbon flow and bacterial communities in aquatic microbial food webs

**DOI:** 10.1073/pnas.2102225118

**Published:** 2021-05-31

**Authors:** Isabell Klawonn, Silke Van den Wyngaert, Alma E. Parada, Nestor Arandia-Gorostidi, Martin J. Whitehouse, Hans-Peter Grossart, Anne E. Dekas

**Affiliations:** ^a^Department of Earth System Science, Stanford University, Stanford, CA 94305;; ^b^Department of Experimental Limnology, Leibniz-Institute of Freshwater Ecology and Inland Fisheries, 12587 Berlin, Germany;; ^c^Department of Geosciences, Swedish Museum of Natural History, 104 05 Stockholm, Sweden;; ^d^Institute of Biochemistry and Biology, Potsdam University, 14476 Potsdam, Germany

**Keywords:** eukaryotic microparasites, phytoplankton–fungi–bacteria interactions, carbon fluxes

## Abstract

Microbial interactions in aquatic environments profoundly affect global biogeochemical cycles, but the role of microparasites has been largely overlooked. Using a model pathosystem, we studied hitherto cryptic interactions between microparasitic fungi (chytrid *Rhizophydiales*), their diatom host *Asterionella,* and cell-associated and free-living bacteria. We analyzed the effect of fungal infections on microbial abundances, bacterial taxonomy, cell-to-cell carbon transfer, and cell-specific nitrate-based growth using microscopy (e.g., fluorescence in situ hybridization), 16S rRNA gene amplicon sequencing, and secondary ion mass spectrometry. Bacterial abundances were 2 to 4 times higher on individual fungal-infected diatoms compared to healthy diatoms, particularly involving Burkholderiales. Furthermore, taxonomic compositions of both diatom-associated and free-living bacteria were significantly different between noninfected and fungal-infected cocultures. The fungal microparasite, including diatom-associated sporangia and free-swimming zoospores, derived ∼100% of their carbon content from the diatom. By comparison, transfer efficiencies of photosynthetic carbon were lower to diatom-associated bacteria (67 to 98%), with a high cell-to-cell variability, and even lower to free-living bacteria (32%). Likewise, nitrate-based growth for the diatom and fungi was synchronized and faster than for diatom-associated and free-living bacteria. In a natural lacustrine system, where infection prevalence reached 54%, we calculated that 20% of the total diatom-derived photosynthetic carbon was shunted to the parasitic fungi, which can be grazed by zooplankton, thereby accelerating carbon transfer to higher trophic levels and bypassing the microbial loop. The herein termed “fungal shunt” can thus significantly modify the fate of photosynthetic carbon and the nature of phytoplankton–bacteria interactions, with implications for diverse pelagic food webs and global biogeochemical cycles.

## Significance

Planktonic microorganisms interact with each other in multifarious ways, ultimately catalyzing the flow of carbon and energy in diverse aquatic environments. However, crucial links associated with eukaryotic microparasites are still overlooked in planktonic networks. We addressed such links by studying cryptic interactions between parasitic fungi, phytoplankton, and bacteria using a model pathosystem. Our results demonstrate that parasitic fungi profoundly modified microbial interactions through several mechanisms (e.g., transferring photosynthetic carbon to infecting fungi, stimulating bacterial colonization on phytoplankton cells, and altering the community composition of bacteria and their acquisition of photosynthetic carbon). Hence, fungal microparasites can substantially shape the microbially mediated carbon flow at the base of aquatic food webs and should be considered as crucial members within plankton communities.

Parasitism is one of the most common consumer strategies on Earth (1–3). Recently, it has also been identified as one of the dominating interactions within the planktonic interactome (4, 5), and yet parasites remain poorly considered in analyses of trophic interactions and element cycling in aquatic systems (6, 7). The foundation of trophic interactions in plankton communities is set by single-cell phytoplankton, which contributes almost half of the world’s primary production (8). According to our common understanding, the newly fixed carbon (C) is channeled either through the microbial loop, classical food web, or viral shunt, which supports the growth of heterotrophic bacteria and nanoflagellates, zooplankton and higher trophic levels, or viruses, respectively (9). However, fungi, particularly fungal microparasites, are rarely considered as contributors to C and nutrient cycling, although they are present and active in diverse aquatic environments (10–12).

Members of the fungal division Chytridiomycota, referred to as chytrids, can thrive as microparasites on phytoplankton cells in freshwater (11, 13, 14) and marine systems (15–17), infecting up to 90% of the phytoplankton host population (18–21). A recent concept, called mycoloop, describes parasitic chytrids as an integral part of aquatic food webs (22). Energy and organic matter are thereby transferred from large, often inedible phytoplankton to chytrid zoospores, which are consumed by zooplankton (23–27). Hence, parasitic chytrids establish a novel trophic link between phytoplankton and zooplankton. Our understanding of element cycling and microbial interactions during chytrid epidemics, however, remains sparse. For instance, the cell-to-cell C transfer from single phytoplankton cells to their directly associated chytrids has not been quantified to date. Moreover, the relationship between parasitic chytrids and heterotrophic bacteria is largely undescribed.

Phytoplankton cells release substantial amounts of dissolved organic C (DOC) (28), whereby up to 50% of photosynthetic C is consumed as DOC by bacteria (29–32). Thus, bacterial communities are intimately linked to phytoplankton abundances and production (33). Phytoplankton–bacteria interactions are particularly strong within the phycosphere, the region immediately surrounding individual phytoplankton cells (33–35), where nutrient concentrations are several-folds higher compared to the ambient water (36, 37), and nutrient assimilation rates of phytoplankton-associated bacteria are at least twice as fast as those of their free-living counterparts (38, 39). Importantly, parasitic chytrids may distort these phytoplankton–bacteria interactions within and outside the phycosphere since they modulate substrate and nutrient availabilities and presumably also bacterial activity and community composition. The effects of this distortion are virtually unresolved, but the few available data indicate that chytrid infections alter the composition and concentration of DOC (40), while abundances of free-living bacteria increase (25, 40) or remain unchanged (24).

To disentangle phytoplankton–fungi–bacteria interactions at a microspatial single-cell scale—the scale at which phytoplankton, fungi, and bacteria intimately interact—we used one of the few existing model pathosystems, composed of the freshwater diatom *Asterionella formosa*, the chytrid *Rhizophydiales* sp., and coenriched populations of heterotrophic bacteria. Our methodology included dual stable-isotope incubations (^13^C-bicarbonate and ^15^N-nitrate), single-cell–resolution secondary ion mass spectrometry (SIMS) (IMS 1280 and NanoSIMS 50L), 16S rRNA gene/16S rRNA sequencing, microscopy (e.g., fluorescence in situ hybridization [FISH]), and nutrient analyses. We particularly focused on the initial C transfer from the phytoplankton host to parasitic chytrids, which we term the “fungal shunt,” as part of the mycoloop. The objectives were twofold: 1) quantifying the transfer of photosynthetic C from phytoplankton cells to infectious chytrids, cell-associated bacteria, and free-living bacteria and 2) characterizing the effect of parasitic fungi on bacterial populations, considering bacterial abundances, bacterial–diatom attachment, single-cell activity rates, and community composition. The obtained data challenge the common perception of aquatic microbial food webs by demonstrating the significant role that parasitic fungi can play in microbial community structure, interactions, and element cycling during phytoplankton growth.

## Results

### Characterization of the Cocultures.

**Fig. 1.**
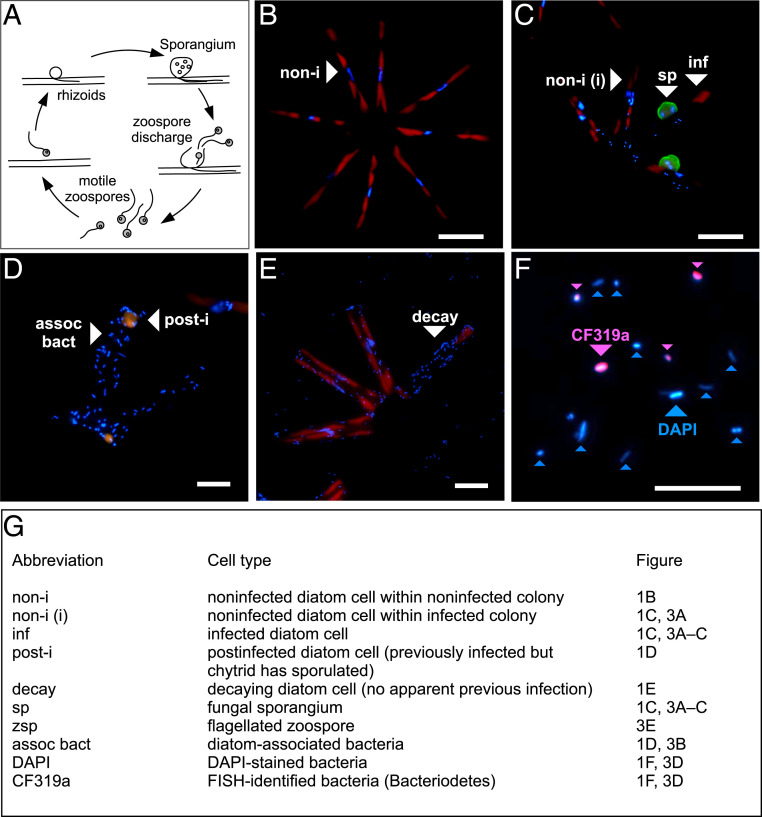
Cell types in the model pathosystem. (*A*) Schematic life cycle (asexual) of parasitic chytrids. During phytoplankton blooms, chytrids replicate quickly by asexual reproduction. Free-living, motile zoospores settle onto a phytoplankton cell, encyst, and expand into the host’s interior via rhizoids. New zoospores are produced in each sporangium and eventually discharged into the water to seek out a new host, leaving behind a dead phytoplankton cell. (*B*–*F*) Fluorescence microscopy images of the diatom *Asterionella*, the parasitic chytrid *Rhizophydiales,* and bacterial cells. *B*–*E* show *Asterionella* and *Asterionella*-associated cells, and *F* shows free-living bacteria (turquoise = DAPI-stained bacteria, pink = CF319a-hybridized cells [i.e., Bacteroidetes]). Chitinous cell walls of the chytrid *Rhizophydiales* in *C* were stained with WGA (conjugated to Alexa Fluor 488). (White scale bars, 10 µm.) (*G*) Description of the various cell types. A higher-quality figure is available on Figshare (https://doi.org/10.6084/m9.figshare.14614155).

Our experiments included stable-isotope incubations of noninfected and chytrid-infected diatom cocultures—referred to as noninfected and infected treatment, respectively. Both cultures included the colony-forming freshwater diatom *A. formosa* (star-like colonies composed of 4 to 10 individual cells) and mixed populations of cell-associated and free-living bacteria (Fig. 1). On day 0, the designated infected treatment was inoculated with the chytrid *Rhizophydiales* sp., which was present as sessile *Asterionella*-associated sporangia and free-living zoospores, both being part of the asexual life cycle of chytrids (Fig. 1*A*) (41–43). The noninfected treatment remained without chytrid inoculum. Both cultures were subsampled on day 0, 2, and 6, during which *Asterionella* abundances increased to 1.5 ± 0.2 × 10^5^ and 1.3 ± 0.4 × 10^5^ cells ⋅ mL^−1^ in noninfected and infected cultures, respectively (*P* = 0.50, *SI Appendix*, Fig. S1 and Table S1). In the infected treatment, up to 27% of the *Asterionella* population was infected by day 6. Since infection prevalence was highest on that day, we present data from day 6 herein, unless stated otherwise.

Chlorophyll *a* concentrations (∼30 to 90 ng ⋅ mL^−1^) were similar in both treatments throughout the entire 6-d period, but phaeopigments (∼2 to 15 ng ⋅ mL^−1^) were significantly higher on day 6 in the infected treatment (see *SI Appendix*, Table S2 for statistics). Nutrient conditions were replete in both treatments (350 to 420 nmol ⋅ mL^−1^ nitrate and ∼35 nmol ⋅ mL^−1^ soluble reactive phosphorous [SRP]). Concentrations of dissolved silica (∼0.2 to 1.6 nmol ⋅ mL^−1^) were comparable on day 0 and 2 but significantly higher in the infected treatment on day 6. Total DOC concentrations (∼70 to 140 nmol ⋅ mL^−1^) were equal in both treatments on all sampling days, but on day 6, the ^13^C enrichment in the DOC pool was significantly higher in the infected treatment (4.0 ± 0.4 versus 4.9 ± 0.0 ^13^C atom percent [atom%] excess, *P* = 0.0001). DOC production rates were similar in both treatments (∼20 nmol C ⋅ d^−1^ ⋅ mL^−1^, *SI Appendix*, Table S2).

### Bacterial Abundances, Community Composition, and Potential Activity.

**Fig. 2.**
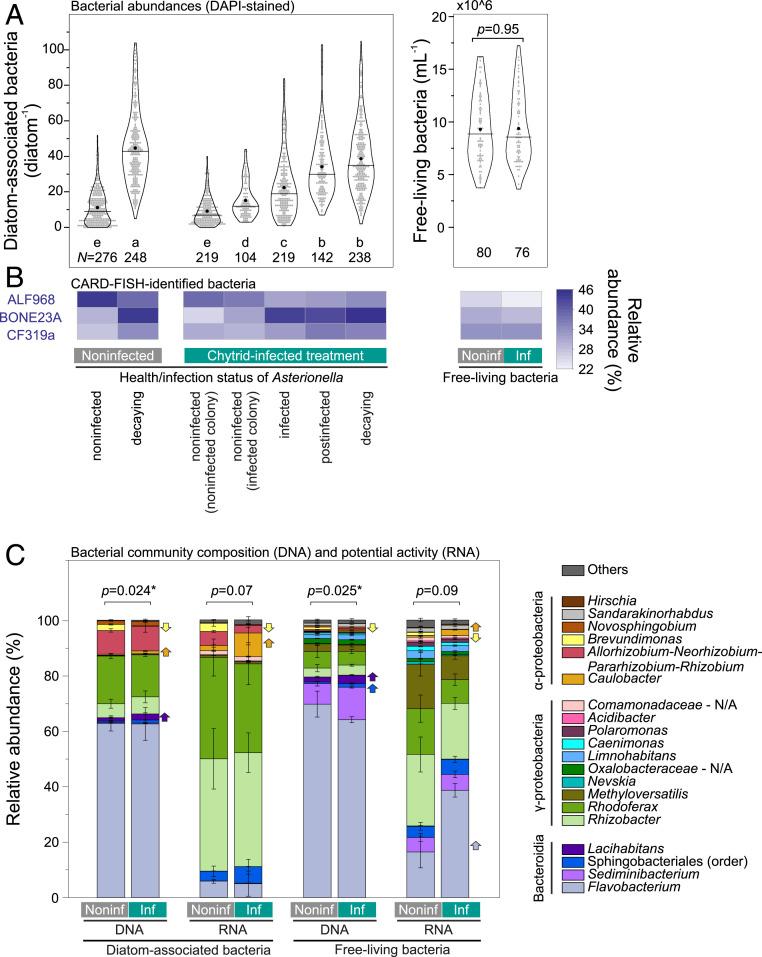
Bacterial abundances and community compositions. (*A*) Abundances of diatom-associated and free-living bacteria. Diatom-associated bacteria were counted on individual *Asterionella* cells of different health/infection status. The letters a through e denote significantly different groups (Kruskal–Wallis, *P* < 0.05). For free-living bacteria, the significant difference is given as *P* value (Mann–Whitney test). The numbers of replicates *N* (*Asterionella* cells or fields of view) are displayed. The vertical lines represent medians and the black circles represent means. (*B*) Relative abundances of major bacterial classes counted as FISH-identified cells (ALF968—Alphaproteobacteria, BONE23A—Gammaproteobacteria, and CF319a—Bacteroidia). The data shown in *A* and *B* are listed in *SI Appendix*, Table S1. (*C*) Bacterial community composition (16S rRNA gene based [i.e., DNA]) and potential activity (16S rRNA based [i.e., RNA]) shown as the relative abundance of ASV counts. Genus taxonomy is given, except for the order Sphingobacteriales (family *env.OPS17*) and the families *Comamonadaceae* and *Oxalobacteraceae*, which did not have assigned genera (N/A). Statistical differences between the overall ASV composition in the noninfected versus chytrid-infected treatment are indicated as *P* values (derived from permutational ANOVA comparing weighted UniFrac distance metrics, asterisks indicate statistically significant differences, *P* < 0.05). The arrows mark taxa with higher or lower ASV counts in the noninfected versus chytrid-infected treatment (*P* < 0.01). The taxa details are listed in *SI Appendix*, Tables S4 and S5. (*A*–*C*) The data are shown for bacteria sampled 6 d after chytrid inoculation (27% infection prevalence). Sequencing data from day 0 and 2 are shown in *SI Appendix*, Fig. S2.

Using fluorescence microscopy, we classified bacteria as 1) free-living bacteria and diatom-associated bacteria based on their attachment to different types of *Asterionella* cells, including 2) noninfected, 3) infected, 4) postinfected, and 5) decaying cells (Fig. 1). Noninfected *Asterionella* were recognized as healthy, intact cells; infected *Asterionella* showed ongoing infections with a mature epibiotic zoosporangium (herein sporangium); postinfected *Asterionella* displayed remains of chytrid-related chitin structures as a sign of previous infections after zoospore discharge; and decaying cells showed low levels of autofluorescence but no signs of previous infections, and thus they were most likely not involved in chytrid infections. The abundance of diatom-associated bacteria was lowest in conjunction with noninfected *Asterionella* (9.2 ± 7.6 bacteria diatom^−1^) and increased on infected, postinfected, and decaying *Asterionella* (22.4 ± 16.7, 34.1 ± 17.4, and 38.7 ± 19.2 bacteria diatom^−1^, respectively, Fig. 2*A*). Moreover, in the infected treatment, we distinguished between noninfected colonies (zero infections, Fig. 1*B*) and infected colonies (at least one infected *Asterionella* cell in addition to noninfected cells, Fig. 1*C*). Noninfected *Asterionella* cells within infected colonies were more heavily colonized by bacteria than noninfected *Asterionella* cells within noninfected colonies (15.2 ± 9.7 versus 9.2 ± 7.6 bacteria diatom^−1^). In total, bacteria associated with post/infected *Asterionella* comprised 43% of the diatom-associated population although only 27% of the *Asterionella* population was post/infected. In the free-living community, bacterial abundances were not significantly different between treatments (∼9 × 10^6^ bacteria mL^−1^, *P* = 0.95, Fig. 2*A*). See *SI Appendix*, Table S1 for detailed numbers and statistics of diatom and bacterial abundances (including FISH-identified cells, described below).

Bacterial communities of *Asterionella*-associated and free-living bacteria (size-fractionated after gentle filtration and analyzed via 16S rRNA gene sequencing) were composed mostly of Bacteroidetes, (59 to 86%), followed by Gammaproteobacteria (11 to 26%) and Alphaproteobacteria (2 to 15%, ranges reflect the variability across both *Asterionella*-associated and free-living populations, Fig. 2*C*). By contrast, 16S rRNA reads [a proxy for potential protein synthesis (i.e., potential activity) (44)] were dominated by Gammaproteobacteria (42 to 88%) and to a lesser extent by Bacteroidetes (4 to 52%) and Alphaproteobacteria (2 to 19%). On a genus level, *Allorhizobium*–*Neorhizobium*–*Pararhizobium*–*Rhizobium* (Alphaproteobacteria) was proportionally overrepresented in diatom-associated bacteria (∼9% of the 16S rRNA gene reads) compared to free-living bacteria (0.5%). Contrariwise, the genera/families *Sediminibacterium* (Bacteriodetes), *Sandarakinorhabdus* (Alphaproteobacteria), *Methyloversatilis*, *Oxalobacteraceae*, *Limnohabitans*, *Caenimonas*, and *Polaromonas* (all Gammaproteobacteria) were present in the free-living population (0.4 to 12% of the 16S rRNA gene reads) but virtually absent in the diatom-associated population (0 to 0.3%, ranges reflect different genera/families).

The bacterial community composition in infected cultures had significantly diverged from that in noninfected cultures 6 d after chytrid inoculation (*P* ≤ 0.025), whereas potential activities were not statistically different (*P* ≥ 0.07, PERMANOVA based on weighted UniFrac distances, Fig. 2*C* and *SI Appendix*, Fig. S2). Several taxa demonstrated statistically significant differences (*P* < 0.01) in their relative abundances and potential activities between treatments, including *Lacihabitans*, *Flavobacterium*, Sphingobacteriales (Bacteroidetes), and *Caulobacter* (Alphaproteobacteria), which all showed an increase in their amplicon sequence variant (ASV) counts upon chytrid infection, and *Brevundimonas* (Alphaproteobacteria), whose ASV counts decreased (Fig. 2*C* and *SI Appendix*, Table S5).

Based on the 16S rRNA gene–derived bacterial community composition, we chose three previously designed FISH probes to determine abundances of Bacteriodetes (probe CF319a), Gammaproteobacteria [probe BONE23A targeting Burkholderiales, formerly Betaproteobacteria (45)], and Alphaproteobacteria (probe ALF968). These FISH-identified classes showed rather similar relative abundances (22 to 46%, Fig. 2*B*, statistics are given in *SI Appendix*, Table S1), whereas 16S rRNA gene reads indicated substantially higher proportions of Bacteriodetes compared to Gamma- and Alphaproteobacteria (see above). FISH-identified Gammaproteobacteria were more abundant on post/infected and decaying *Asterionella* cells (43 to 46% relative abundance) compared to noninfected *Asterionella* (25 to 32%, *P* < 0.05, ranges reflect different *Asterionella* types in both treatments). A similar trend was observed for Bacteroidetes associated with post/infected and decaying *Asterionella* cells (32 to 36%) and noninfected *Asterionella* (28 to 31%), but this trend was not statistically significant (*P* > 0.05). In contrast, Alphaproteobacteria were more abundant on noninfected *Asterionella* cells (37 to 45%) than on post/infected and decaying cells (31 to 38%). In the free-living community, Bacteroidetes were most abundant (33% relative abundance), with no statistically significant difference between treatments, while Alphaproteobacteria and Gammaproteobacteria were less abundant in the infected versus noninfected culture (22 versus 26% and 28 versus 31%, respectively, Fig. 2*B*, statistics are included in *SI Appendix*, Table S1).

### Cell-Specific C Transfer and Nitrogen-Based Growth Rates.

**Fig. 3.**
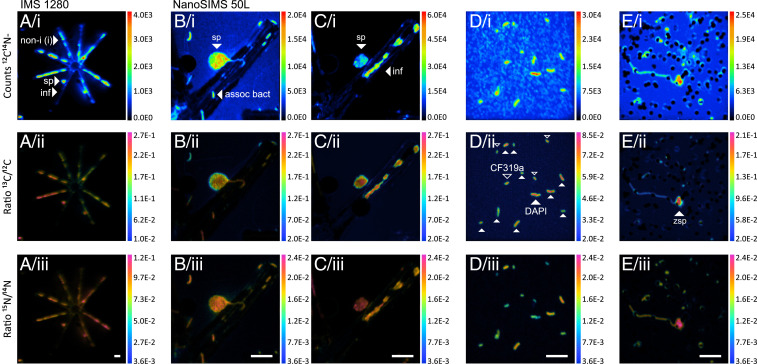
SIMS images. (*A*) *Asterionella* colony with a fungal sporangium analyzed on the IMS 1280 (90 × 90 µm raster). (*B*–*D*) Single *Asterionella* cell, *Asterionella*-associated and free-living bacteria, and (*E*) zoospores analyzed on the NanoSIMS (25 × 25 µm raster). *B* and *C* display the same field of view, first, imaged after 90-s presputtering to analyze diatom-associated bacteria (*B*) and second, after 20-min presputtering to analyze *Asterionella* and its associated sporangium (*C*). Free-living bacteria in *D* correspond to the ones shown in Fig. 1*F*. The filled arrows mark DAPI-stained cells, and the no-filled arrows mark CF319a-hybridized cells (Bacteroidetes). The abbreviations are defined in Fig. 1*G*. (White scale bars, 5 µm [*Bottom*].)

Using stable-isotope tracer incubations (^13^C-dissolved inorganic C [i.e., ^13^C-DIC] and ^15^N-nitrate) in combination with SIMS, we quantified the cell-to-cell transfer of photosynthates and cell-specific N-based growth rates. We did not detect any sequences of autotrophic cyanobacteria in the 16S rRNA gene dataset or any autofluorescent bacteria under the microscope. Moreover, heterotrophic CO_2_ assimilation is unlikely or minimal under nutrient-replete conditions (46). *Asterionella* was thus expected to represent the only C-fixing organism that utilized the supplied ^13^C-DIC, while any ^13^C enrichment in fungal and bacterial cells was due to secondary C transfer from *Asterionella*. In contrast, ^15^N-nitrate was bioavailable to all cells via direct uptake. Therefore, ^15^N-enrichments reflected cell-specific N growth rates based on nitrate assimilation. SIMS-targeted cells included *Asterionella*, *Asterionella*-associated sporangia, and free-swimming zoospores, as well as cell-associated and free-living bacteria (Fig. 3). For free-living bacteria, we distinguished cells as Alphaproteobacteria, Gammaproteobacteria, and Bacteroidetes by prior identification with FISH.

**Fig. 4.**
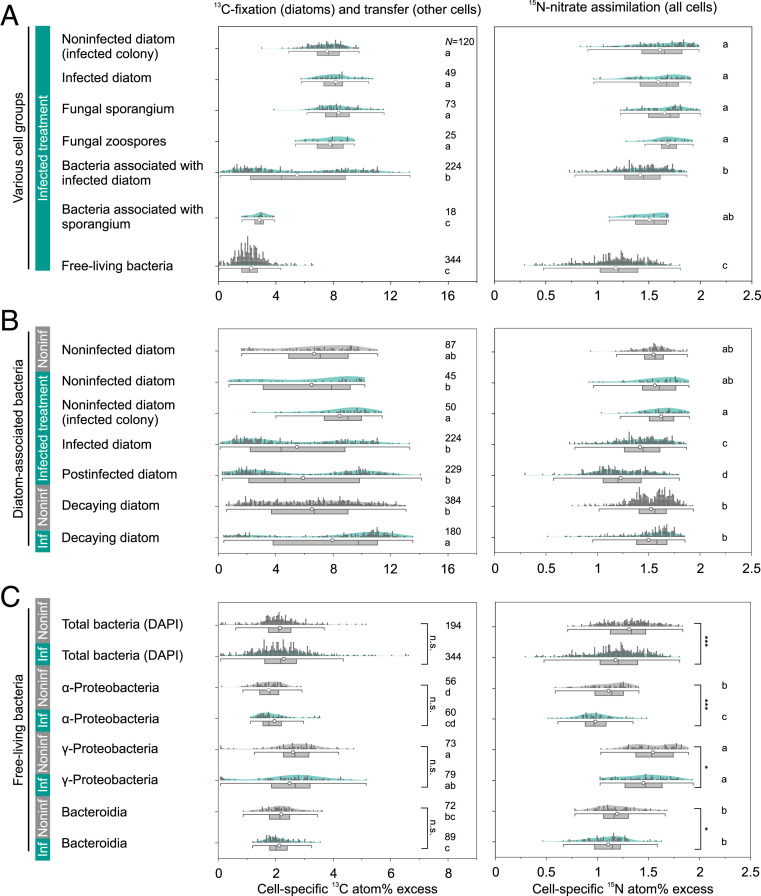
Cell-specific isotope atom% excess in various cell types in the infected treatment (*A*), as well as in diatom-associated and free-living bacteria in both treatments (*B* and *C*). Significant differences are shown as letters (Kruskal–Wallis test, *P* < 0.05, ran separately for ^13^C and ^15^N and separately for cells shown in *A*–*C*). The significant differences of FISH-identified bacteria between both treatments are additionally indicated as n.s. (not significant); *P < 0.05 and ***P < 0.001 (Mann–Whitney test). *N* denotes the number of analyzed cells.

In infected cultures, ^13^C enrichments were higher for noninfected and infected diatoms, their associated sporangia, and zoospores (7.2 ± 1.1, 8.2 ± 1.2, 8.4 ± 1.3 and 7.8 ± 1.1 atom% excess, respectively) than for bacteria (Fig. 4 and *SI Appendix*, Table S3). Among cell-associated bacteria, ^13^C enrichments were lower in bacteria that were involved in chytrid infections (colonizing infected and postinfected *Asterionella* cells and fungal sporangia; 5.5 ± 3.6, 5.9 ± 3.9, and 2.9 ± 0.6 atom% excess, respectively) compared to bacteria not involved with fungal infections (colonizing noninfected and decaying *Asterionella*; 6.5 ± 3.3 and 8.0 ± 4.0 atom% excess, respectively). Moreover, the presence of chytrids induced a split in bacterial subpopulations. That is, in the noninfected treatment, bacteria on decaying diatoms displayed a uniform data distribution across the range of ^13^C enrichments (Fig. 4*B*). By contrast, in the infected treatment, bacteria on post/infected and decaying diatoms showed a bimodal data distribution, with the over-representation of one bacterial subpopulation of low ^13^C enrichment on post/infected *Asterionella* and one subpopulation of high ^13^C enrichment overrepresented on decaying *Asterionella*. The lowest ^13^C and ^15^N enrichments were found in free-living bacteria (range of means: 1.8 to 2.6 ^13^C atom% excess and 1.0 to 1.5 ^15^N atom% excess), of which Gammaproteobacteria were more enriched than Alphaproteobacteria and Bacteroidetes. For each free-living bacterial class, ^13^C enrichments were not significantly different between treatments, but ^15^N enrichments were significantly lower in the infected treatment (see Fig. 4*C* for data distributions and statistics and *SI Appendix*, Table S3 for detailed values for all cell types, including N-based growth rates presented below).

Mean N-based growth rates (SIMS derived, based on cell-specific ^15^N-nitrate assimilation) were higher for *Asterionella*, chytrid zoosporangia, and zoospores (0.19 to 0.23 d^−1^) than for cell-associated and free-living bacteria (0.12 to 0.19 d^−1^ and 0.09 to 0.18 d^−1^, respectively, Fig. 4). N-based growth rates decreased from bacteria associated with noninfected and decaying diatoms (0.17 to 0.19 d^−1^) to those associated with post/infected diatoms (0.12 to 0.15 d^−1^). For *Asterionella*, cell-specific N-based growth rates (0.19 to 0.23 d^−1^, derived from SIMS) compared well with their population-specific growth rates (0.21 to 0.24 d^−1^, derived from cell counts). The population-specific growth of free-living bacteria was 0.26 to 0.27 d^−1^ (i.e., faster than implied by only nitrate assimilation). The population-specific growth of sporangia was 0.64 d^−1^, exceeding the growth of *Asterionella*.

**Fig. 5.**
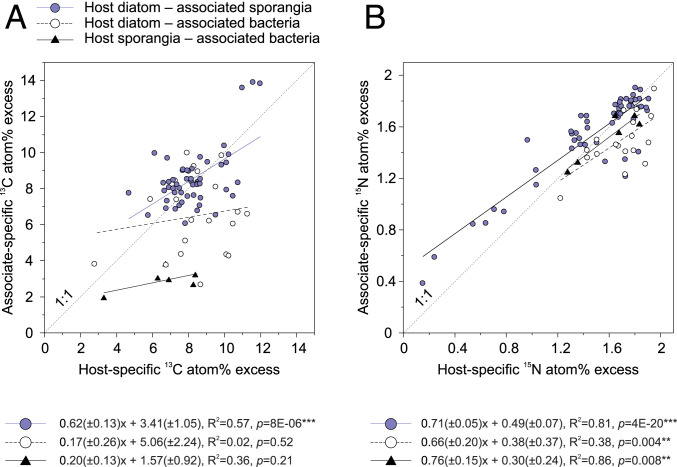
Correlation between isotope ratios ([*A*] ^13^C and [*B*] ^15^N atom% excess) measured for host cells (diatom or fungi) and their directly associated cells (fungi or bacteria). Each data point represents the mean ratio of one host cell and its multiple associates (*n* = 1 to 34). The results of linear regression are shown. The slopes were significantly different from zero at *P* < 0.05 (**P < 0.01 and ***P < 0.001).

In some instances, we were able to analyze ^13^C and ^15^N isotope ratios in host cells (*Asterionella* or sporangia) and their direct host-associated cells (sporangia or bacteria). The ^13^C atom% excess in *Asterionella* and their associated sporangia followed a trend close to a 1:1 ratio, with a slope of linear regression being significantly different from zero (*R*^2^ = 0.57, *P* = 8 × 10^−6^, Fig. 5*A*). The ^13^C atom% excess of *Asterionella* and their associated bacteria also scattered close to a 1:1 ratio, but the linear regression was not significant (*P* = 0.52). Sporangia were sparsely colonized by bacteria, and thus we could only analyze six sporangia and their associated bacteria. Nevertheless, these few cells indicated that sporangia were more enriched in ^13^C than their associated bacteria. The ^15^N atom% excess of all host–associate pairings followed a trend close to a 1:1 ratio, with significant linear regression (*R*^2^ = 0.38 to 0.86, *P* ≤ 0.008, Fig. 5*B*).

### C Transfer within the Model Pathosystem.

**Fig. 6.**
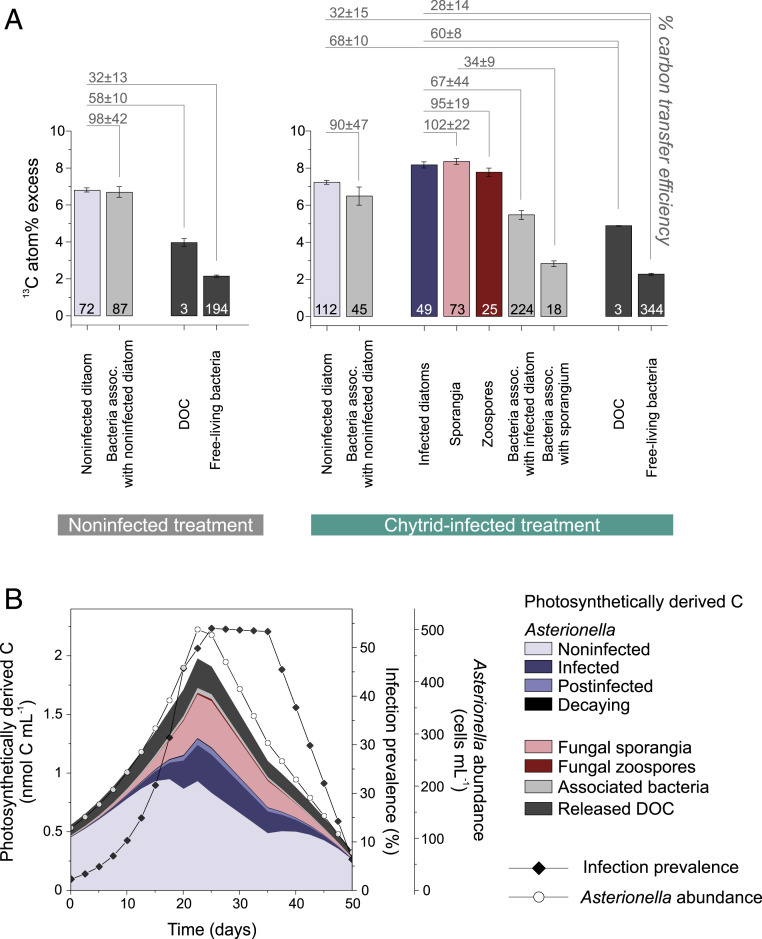
Partitioning of photosynthetic C in single-cell populations (*A*) and whole cell communities (*B*). (*A*) Mean ^13^C atom% excess (±SE) of various cell populations and the DOC pool. The number of replicate cells (or incubation flasks for DOC) is given for each bar. The C transfer efficiency was calculated as the percent of ^13^C enrichment in a given cell type (i.e., sporangia, bacteria, or the DOC pool) relative to the ^13^C enrichment in its source cell (i.e., diatoms or sporangia). (*B*) Distribution of photosynthetically derived C extrapolated for a naturally grown *Asterionella* population during an infection prevalence of up to 54% over 50 d (stacked areas). The lines show *Asterionella* abundances and infection prevalences (both derived from ref. 47).

By quantifying cell-specific ^13^C incorporation, we were able to track the C transfer from phototrophic *Asterionella* to cogrowing chytrids and bacteria. As an index of C transfer efficiency, we calculated the percent of ^13^C enrichment in a given cell type (i.e., sporangia, bacteria, or the DOC pool) relative to the ^13^C enrichment in its source cell (i.e., diatoms or sporangia). The C transfer efficiency from diatoms to sessile sporangia was 102%, to free-swimming zoospores 95%, to *Asterionella*-associated bacteria 67 to 98%, to free-living bacteria 28 to 32%, and to the DOC pool 58 to 68% (Fig. 6*A*, the percentage for sporangia exceeding 100% might be explained by the high cell-to-cell variability in ^13^C enrichments within cell populations). The transfer efficiency from sporangia to their associated bacteria was 34%. Bacteria associated with healthy *Asterionella* cells acquired, on average, relatively more photosynthetic C than those associated with chytrid-infected *Asterionella* (90 to 98% versus 67% C transfer efficiency, Fig. 6*A*). We further followed the fate of diatom-derived C within individual noninfected versus chytrid-infected *Asterionella* and its associated cells (not including any transfer to the ambient water). In noninfected *Asterionella* cells, 99% of the diatom-derived C was maintained in the *Asterionella* cell, while 1% was transferred to its associated bacteria. By comparison, in infected *Asterionella* cells, the diatom cell maintained only 45% of its C biomass, 53% was directed to the infecting sporangia, and 2% to associated bacteria.

### C Transfer within a Natural Plankton Population.

To extrapolate the partitioning of photosynthetically derived C between microplankton groups in a natural community, we applied our quantitative single-cell data to literature-derived data of a chytrid-infected *Asterionella* population (*Asterionella* abundance and infection prevalence) in Lake Maarsseveen (Netherlands) during spring 2009 (47). For this natural *Asterionella* population, we estimate that 20% of the photosynthetically derived C was transmitted to the chytrid population at the prevalence peak (54% infection prevalence, Fig. 6*B*). The *Asterionella* population retained 59% of the photosynthetically derived C biomass, cell-associated bacteria received 2%, and 19% was released as DOC and thus available to free-living bacteria. In contrast, in a scenario without chytrid infections, *Asterionella* populations would have retained 75% of the photosynthetic C, 1% would have been incorporated by *Asterionella*-associated bacteria, and 24% released as DOC.

## Discussion

The first *Asterionella*–chytrid pathosystem was discovered in the mid-20th century in British Lakes (48). Since then, numerous records of parasitic chytrids have been made on a wide range of phytoplankton taxa in freshwater (49–52) and coastal marine waters across various climate zones (16, 17, 53–56). Nevertheless, fungal–phytoplankton parasitism remains poorly considered in microbial networks—owing to the limited availability of empirical data and model systems (11). To shed light on these enigmatic interactions, we studied tripartite cross-kingdom interactions between parasitic fungi, phytoplankton, and cogrowing bacteria. As discussed in the following, these interactions substantially impact microbial communities and the fate of photosynthetic C, as C is channeled through the fungal shunt.

### Parasitic Fungi Modulate Bacterial Abundances and Community Compositions.

*Asterionella* cells infected by fungi were 2 to 4 times more heavily colonized by heterotrophic bacteria, particularly by Burkholderiales and also Bacteroidetes, as compared to noninfected *Asterionella* cells. This promotion in bacterial colonization implies that post/infected cells represented hotspots of leaking nutrient plumes, which commonly encourage bacterial chemotaxis (37, 57–59). As a result, post/infected *Asterionella* showed a more similar colonization pattern to decaying diatoms than to healthy diatoms (Fig. 2). The preferential colonization of FISH-identified Burkholderiales and Bacteroidetes on post/infected and decaying *Asterionella* is consistent with their ability to chemotax toward lysing cells (60, 61). Additionally, those bacterial classes are known as competitive taxa during phytoplankton blooms in lakes and marine systems (62–65), with a copiotrophic, often particle-associated lifestyle (66).

The 16S rRNA gene–derived community compositions of *Asterionella*-associated and free-living bacteria significantly diverged between noninfected and chytrid-infected cultures, as infection prevalence reached 27%, but overall taxonomic compositions remained rather similar, even at the genus level (Fig. 2*C*). Recently, saprotrophic chytrids (as opposed to the parasitic chytrids studied here) were shown to shape field-sampled bacterial communities on artificial chitin beads at the order level within 1 d of inoculation (67), demonstrating that rapid taxonomic shifts are not unprecedented. Abundances of free-living bacteria were similar in both our treatments, in agreement with ref. 24, but during later epidemic stages, other studies have observed two- to fivefold increases in total bacterial abundances (25, 40). Thus, at the herein studied epidemic stage, the presence of chytrids affected abundances and taxonomy of *Asterionella*-associated bacteria and the taxonomy for free-living bacteria. Abundances and taxonomies might have continued to diverge between noninfected and infected communities as the epidemic progressed.

### C Transfer within the Phycosphere during Fungal Infections.

In addition to physical attachment, microbial interactions typically include the exchange of nutrients. Since infected *Asterionella* and epibiotic sporangia showed equal ^13^C enrichments (Figs. 4–6), we conclude that fungal sporangia efficiently siphoned C from the host cell, thereby deriving their entire C content from the diatom host. Thus, fungal rhizoids functioned as direct pipelines of nutrients from the host’s interior to the sporangium on the host’s exterior. Individual sporangia received approximately one-half (53%) of the host’s C biomass. Hence, on average, two sporangia per *Asterionella* cell may develop to its full capacity in the herein-investigated pathosystem. More than two infections may instead lead to smaller sporangia, which were previously found to produce fewer zoospores during late epidemic stages when host abundances are limiting (68). Zoospores also received ∼100% of their cellular C content from diatom-derived photosynthesis. This finding supports the hypothesis that zoospores rely entirely on their endogenous reserves and do not feed after being discharged from the sporangium and while foraging for a new host (69). The efficiency of C transfer from *Asterionella* to their associated bacteria was higher (67 to 98%) than for sporangia-associated bacteria (34%), similar to free-living cells (32%). The incorporation of photosynthates was thus at least twice as efficient at the diatom–water interface as compared to the sporangium–water interface, indicating that chitinous cell walls were effective seals for nutrient leakage, and/or the sporangia-associated bacteria were poor in incorporating fungal-derived C.

The acquisition of photosynthates by *Asterionella*-associated bacteria was highly dependent on the infection status of the diatom cell and colony (Fig. 4*B*). Bacterial colonization and ^13^C incorporation tended to be higher on noninfected *Asterionella* cells within partly infected colonies than on noninfected cells in entirely noninfected colonies, demonstrating that infections affected adjacent seemingly healthy *Asterionella* cells within the same colony, likely due to enhanced enzymatic activity and nutrient provisioning. Bacterial attachment was also promoted on post/infected diatoms; however, those bacteria acquired, on average, fewer photosynthates compared to bacteria on healthy *Asterionella* cells during the 6-d incubation period (67 versus 90 to 98% C transfer efficiency). The cell-to-cell variability in ^13^C incorporation by *Asterionella*-associated bacteria was high, implying different patterns in C utilization of those bacteria and/or different residence times on their diatom host. We propose that chytrid-induced cell lysis of diatoms—as indicated by, for example, high concentrations of phaeopigments as a degradation product of chlorophyll *a*—promoted bacteria that grew opportunistically on the lysing *Asterionella* cells. Healthy *Asterionella* cells and their associated bacteria may instead have engaged in mutualistic relationships. Considering the bimodal distribution of ^13^C enrichments in bacteria that were associated with post/infected *Asterionella* (Fig. 4*B*), we hypothesize that high ^13^C enrichments were related to mutualistic bacteria, which resided on their diatom host on longer terms and thus received photosynthetic C from *Asterionella* effectively during the entire or most of the incubation period. In contrast, low ^13^C enrichments were likely linked to opportunistic bacteria, which colonized *Asterionella* cells during and/or after infection and thus with shorter residence times. Hence, chytrid infections potentially shifted the nature of phytoplankton–bacteria interactions from mutualism to opportunism within the phycosphere, which remains to be confirmed in future studies. We expect that, compared to the herein-used nutrient-replete culture system, the observed shifts in microbial interaction and also taxonomy are even more pronounced in field populations, where microbial communities are more diverse and microbial interactions especially crucial to sustaining growth under in situ, often low-nutrient conditions [e.g., by exchanging N (70), iron (71), sulfur (72), or vitamins (73)].

The nutritional linkage between *Asterionella* and fungal sporangia was the strongest observed due to the most efficient C transfer (Fig. 6*A*) and their synchronized C and N growth (Fig. 5). By contrast, the transfer of photosynthates from *Asterionella* to their associated bacteria was weakly correlated despite synchronized N-based growth rates, likely due to the above-mentioned high cell-to-cell variability. N-based growth rates of *Asterionella*-associated bacteria were up to twice as fast as for their free-living counterparts, although nitrate was replete in the medium for both free-living and cell-associated bacteria. We therefore presume that the growth of diatom-associated bacteria within the phycosphere was promoted by the provision of, for example, sulfur, trace metals, and detoxifying byproducts, besides C and N, as common in diatom–bacteria interactions (72, 74–78).

### The Potential Impact of Parasitic Fungi on DOC.

The *Asterionella* population investigated here released 18% of its newly fixed C as DOC, within the range of 2 to 50% previously reported for phytoplankton in various habitats (28). Population-level DOC production rates were equal with and without infection, but interestingly, the DOC pool was significantly more enriched in ^13^C in the infected culture. We interpret this finding as an enhanced liberation of recently fixed ^13^C into the DOC pool and/or as a shift in DOC composition and bioavailability upon chytrid infections. Indeed, chytrid infections have been demonstrated to lead to less labile (tryptophan-like substances) and more semilabile material (humic-like substances) in the DOC pool (40). In our incubations, DOC concentrations exceeded the bacterial C demand, and thus bacteria could be selective in their DOC-compound assimilation. If we assume that chytrid-infected *Asterionella* released C substrates that were less bioavailable than those released by healthy *Asterionella*, bacteria might have discriminated against those less-bioavailable substrates, potentially leading to the observed higher ^13^C-DOC enrichment in chytrid-infected versus noninfected cultures. This suggestion, however, remains to be validated in future studies.

### Quantifying the Fungal Shunt and Its Comparison to the Microbial Loop and Viral Shunt.

**Fig. 7.**
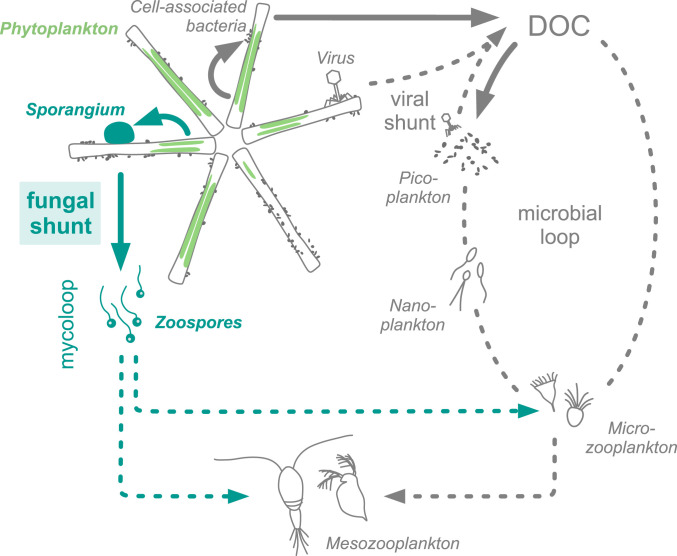
Scheme of C transfer pathways within planktonic food webs. The fungal shunt (solid, turquoise arrows) diverts phytoplankton-derived C to chytrid sporangia and their free-swimming zoospores, promoting C transfer to higher trophic levels and bypassing the microbial loop (schematically shown as dashed ellipse) and viral shunt. The fungal shunt together with the subsequent consumption of zoospores by micro- and mesozooplankton are described as the mycoloop. The solid arrows indicate pathways investigated in this study. For simplicity purposes, the scheme does not embrace the full complexity of C transfer pathways between the shown plankton groups.

For a natural lacustrine system, we estimated that 20% of the photosynthetically derived C was directed to chytrids during an epidemic with 54% infection prevalence (Fig. 6*B*). This percentage conforms with data derived from large-scale food web modeling, suggesting that 19 to 21% of primary production is transferred from phytoplankton to parasitic chytrids (79, 80). In the model, the authors used a mesoscale mass balance approach, while we provide empirical data directly quantifying cell-to-cell C transfer rates between phytoplankton, parasitic chytrids, and bacteria. Remarkably, the fungi-mediated C flow is similar in magnitude to those through the microbial loop or viral shunt. The microbial loop shuttles 10 to 50% of photosynthetic C from primary producers to heterotrophic bacteria, which can be subsequently grazed by flagellates or ciliates, recycled as DOC, or respired back to inorganic C (32). The viral shunt has been estimated to liberate 2 to 10% of photosynthetic C to the DOC pool during viral-induced cell lysis (81), thereby stimulating C transfer from phytoplankton to free-living bacteria (82) and thus microbe-mediated remineralization (83, 84). As opposed to the viral shunt, we suggest that parasitic chytrids shunt photosynthetic C to the particulate organic C pool since we demonstrated that C is most efficiently transferred to attached sporangia, and the therein developed zoospores, which, as shown previously, are grazed by micro- and mesozooplankton (23–27). Moreover, chytrid infections accelerated diatom decay, stimulating bacterial colonization (by potentially opportunistic bacteria that thrive on decaying organic particles). However, chytrids reduced the overall photosynthetically active biomass and thus also the phytoplankton-derived contribution to the DOC pool. For instance, at an infection prevalence of 54%, this contribution decreased from 24 to 19%. We term this fungal-induced diversion of photosynthetic C from the microbial loop to parasitic chytrids the fungal shunt, as part of the previously described mycoloop (22). In this vein, we propose that the fungal shunt promotes zooplankton-mediated over microbe-mediated remineralization (Fig. 7).

Our results demonstrate that parasitic fungi modulate phytoplankton–bacteria interactions and divert significant fractions of photosynthetically derived C from the classically understood microbial loop. Hence, given that parasitic chytrids are distributed in highly relevant aquatic systems, such as areas impacted by harmful algae blooms (55, 85), commercial mass cultures (86), and productive upwelling regions (15), these interactions need to be integrated into future considerations of microbial networks and biogeochemical cycles in the aquatic environment.

## Materials and Methods

### Experimental Setup.

The model pathosystem comprised the diatom host *A. formosa* and the parasitic chytrid *Rhizophydiales* sp., isolated from Lake Stechlin (Northern Germany) in December 2016. Bacteria were coisolated with the diatom–chytrid system and maintained in coculture in nutrient-replete medium for several months, which likely selected for copiotrophic taxa. Batch cultures, comprising *Asterionella*, the chytrid, and bacteria, were grown in CHU-10 medium at constant temperature (17 °C). The light regime was 16:8 h, providing 40 µE ⋅ s^−1^ ⋅ m^−2^ during the 16-h light phase. *Asterionella* was pregrown until entering the early exponential phase (∼3.6 × 10^4^ cells ⋅ mL^−1^) and thereafter split as 8 × 650 mL into 1-L Erlenmeyer flasks. Of those, 6 × 650 mL were amended with isotope tracers, while 2 × 650 mL were left without isotope amendments, serving as controls for later isotope analyses. Isotopes were added as predissolved ^13^C-DIC and ^15^N-nitrate (both Sigma-Aldrich). Subsequently, half of the incubation flasks (3× with isotope addition/1× without) were inoculated with an *Asterionella*–*Rhizophydiales* coculture—referred to as infected treatment. The onset infection prevalence was 2%, including infected diatom cells (with encysted zoospore or mature sporangia) and postinfected cells (with rhizoid remains). The inoculum volume was minor with 8 mL, equal to ∼1% of 650 mL. The additional 4 × 650 mL (3× with isotope addition/1× without) were left without *Rhizophydiales*—referred to as noninfected treatment—but we added 8 mL of nonlabeled medium to account for the isotope dilution in the infected treatment. Each replicate was subsampled on day 0 and after 2 and 6 d, except for samples for raw fluorescence, which were taken daily.

### Culture Characterization.

Subsamples were taken for the following parameters and analyses: 1) raw fluorescence and pigment analyses (chlorophyll *a* and phaeopigments) via fluorometry, 2) enumeration of diatoms, zoospores, sporangia, and bacteria via microscopy, 3) bacterial community analyses by 16S rRNA gene/16S rRNA amplicon sequencing and FISH, 4) single-cell isotope analyses via SIMS, 5) bulk isotope analyses (^13^C-DIC, ^13^C-DOC, ^13^C-particulate organic C/^15^N-particulate organic N, and ^15^N-nitrate) using mass spectrometry, and 6) nutrient analyses by flow analysis and spectrometry (nitrate, SRP, and dissolved silica) or combustion catalytic oxidation (DOC, *SI Appendix*, Table S6). Detailed information on each sampling, analytical procedure, and calculation, as well as complementary data are presented in the *SI Appendix*, while in the following we provide only a short methodological description.

### Nucleic Acid Extraction and Amplicon Sequencing of Bacterial Communities.

Bacteria associated with large diatom colonies (>5 µm) were collected onto 5.0-µm polycarbonate (PC) filters, while free-living bacteria were collected onto 0.2-µm PC filters from the 5.0-µm filtrate (i.e., the water that passed through the 5.0-µm filters). This separation of cell-associated and free-living bacteria may be seen as a snapshot of cell association since some bacteria may alternate between attachment and detachments. DNA and RNA were extracted, and the RNA converted to complementary DNA (cDNA) as previously described (87, 88). The 16S rRNA–encoding DNA and cDNA were amplified using the primer set 515F/926R (V4 to V5 region) (89) following ref. 90. Illumina Sequencing (MiSeq PE250) was performed at the University of California, Davis Genome Center. Amplicon sequences were analyzed following ref. 91 in R (version 3.6.1) (92). The SILVA 138 SSU (small subunit) database was used for assigning taxonomy to each ASV.

### Abundances of Diatoms, Zoospores, Sporangia, and Bacteria.

Samples for diatom, sporangium, and zoospore enumeration were preserved with Lugol and analyzed under an inverted fluorescence microscope (Nikon TiE, 400× magnification). Chitinous cell walls of sporangia were visualized with wheat germ agglutinin (WGA conjugated to Alexa Fluor 488) (93) (Fig. 1*C*). Samples for bacterial enumeration were preserved with paraformaldehyde (1.5% final concentration) and filtered onto PC filters (0.2 µm). Bacterial abundances were determined on filters that were WGA- and 4′,6-diamidino-2-phenylindole (DAPI)-stained, using fluorescence microscopy (1,000×). Furthermore, we conducted catalyzed reported deposition-FISH (CARD-FISH) following refs. 90 and 94 to quantify the major bacterial taxa, as inferred from the sequencing analyses. The probes included: ALF968 (Alphaproteobacteria), CF319a (Bacteroidia), and BONE23A (specific to the gammaproteobacterial order Burkholderiales, formerly Betaproteobacteria). Those probes covered well the entire bacterial community since the FISH-identified cells represented 102 ± 12% of the DAPI-stained bacteria (*n* = 9 different cell types, range: 83 to 117%).

### SIMS.

To quantify the incorporation of ^13^C-DIC and ^15^N-nitrate into single cells (*Asterionella*, sporangia, zoospores, and bacteria), cells were collected onto PC filters and analyzed with two types of CAMECA ion microprobes, the IMS 1280 (at the Natural History Museum, Stockholm) and the NanoSIMS 50L (at the Stanford Nano Shared Facilities). The NanoSIMS offers a higher spatial resolution (∼100 nm) than the IMS 1280 (1,000 nm), but the latter allows for a higher sample throughput, and its higher primary ion beam current facilitates the removal of silica frustules in diatoms. Accordingly, we analyzed diatoms (l × w × h = 20 × 5 × 5 µm) and sporangia (d = 5 µm) preferentially (but not exclusively) on the IMS 1280, whereas bacteria (d = 1 to 2 µm) and zoospores (d = 2 µm) were exclusively analyzed on the NanoSIMS. Analyses were done on cells sampled on day 6, except for some diatoms and sporangia, which were also analyzed from day 2 (data of diatoms and sporangia from day 2 are only included in Fig. 5 to provide a wider range of isotope enrichments, otherwise the data presentation and interpretation are based entirely on cells sampled on day 6). Free-living bacteria were differentiated as Alphaproteobacteria, Gammaproteobacteria, and Bacteroidia using CARD-FISH in combination with NanoSIMS (FISH–SIMS) but we did not target any diatom-associated bacteria via FISH–SIMS since the differentiation between hybridized and nonhybridized cells was ambiguous on the final SIMS images due to an uneven topography of diatom frustules. SIMS analyses were conducted as described previously (90, 95).

### Isotope Ratios, C Transfer, and N-Based Growth Rates.

Single-cell isotope ratios were defined based on the collected ions as: ^15^N/^14^N = ^15^N^12^C^−^/^14^N^12^C^−^ (IMS 1280 and NanoSIMS), ^13^C/^12^C = ^13^C^14^N^−^/^12^C^14^N^−^ (IMS 1280), or ^13^C/^12^C = ^12^C^13^C^−^/^12^C_2_^−^ × 0.5 (NanoSIMS). In the latter, the factor 0.5 is explained by the probability of generating ^12^C_2_^−^ and ^12^C^13^C^−^ ions from ^12^C^−^ and ^13^C^−^. Due to reasons of instrument tuning and higher ion yields, we used the diatomic ^12^C_2_^−^ and ^12^C^13^C^−^ ions instead of the monoatomic ^12^C^−^ and ^13^C^−^ ions for calculating NanoSIMS-derived ^13^C/^12^C ratios (*SI Appendix*, Fig. S3). Ratios were corrected for instrumental mass fractionation using control cells (96) and for isotope dilution during sample preparation using herein-determined dilution factors (97–99) and finally converted to atom% excess (96). The C transfer efficiency from photosynthetic *Asterionella* to other cell groups and the DOC pool was calculated based on the ^13^C atom% excess. To determine cell-specific N-based growth rates, we calculated the fraction of newly incorporated N by single cells relative to their initial N content following ref. 100. For comparison, we also calculated growth rates for entire cell populations (i.e., population-specific growth rates, based on cell counts).

### Extrapolating C Transfer in a Natural System.

To budget C flows in a natural plankton community during a chytrid epidemic, we used data on *Asterionella* populations (*Asterionella* and chytrid abundances) in Lake Maarsseveen (Netherlands) in spring 2009 (47) and combined those data with the herein-determined cell-specific diatom-derived C contents, absolute C contents, DOC production rates, and abundances of diatoms-associated bacteria.

## Supplementary Material

Supplementary File

## Data Availability

The sequence data have been deposited in the European Nucleotide Archive at the European Bioinformatics Institute under accession no. PRJEB44061. A higher-quality Fig. 1 is available on Figshare (DOI: 10.6084/m9.figshare.14614155).
