# Supplementary material for: Characterizing the “fungal shunt”: Parasitic fungi on diatoms affect carbon flow and bacterial communities in aquatic microbial food webs
Source: Proc Natl Acad Sci U S A. 2021 May 31;118(23):e2102225118. doi: 10.1073/pnas.2102225118 (PMC8201943; doi:10.1073/pnas.2102225118)
Supplement: Supplementary File [file pnas.2102225118.sapp.pdf]

SI Appendix

Characterizing the 'fungal shunt': Parasitic fungi on diatoms affect carbon flow and bacterial communities in aquatic microbial food webs

Isabell Klawonn, Silke Van den Wyngaert, Alma E. Parada, Nestor Arandia-Gorostidi, Martin Whitehouse, Hans-Peter Grossart, and Anne E. Dekas

Results

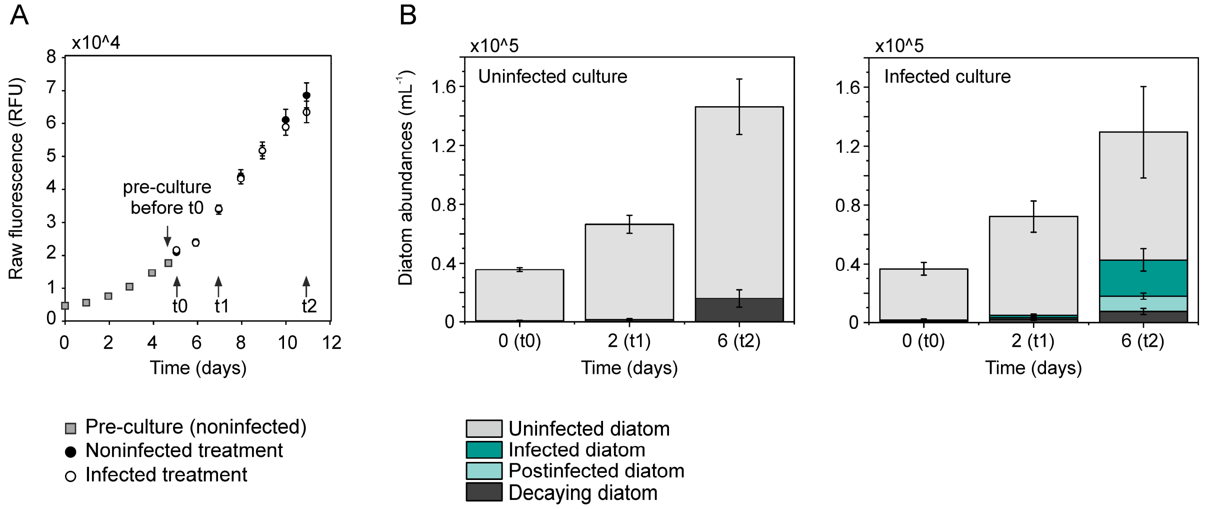

SI Figure S1. (A) Growth curve of *Asterionella* based on raw fluorescence. The culture was grown until reaching the early exponential phase (pre-culture before t0). At t0, stable-isotope tracers were added and the culture was split into two treatments—the noninfected and infected treatment. Samples were taken on day 0, 2, and 6 after chytrid inoculation. **(B)** Diatom abundances during the incubation period (t0–t2). Abundances are shown for noninfected, infected, postinfected, and decaying *Asterionella* cells (mean±SD, *n*=4).

SI Table S1. Diatom and bacteria abundances (plotted in SI Appendix, Fig. S1B and Fig. 2A and B, respectively).

Day	Cell type	Noninfected treatment Mean ± SD	N†	Infected treatment Mean ± SD	N†	Unit	Significance‡	
**Diatom abundances**								
Day 0	Total	$3.6\times10^4\pm1.8\times10^3$	4	$3.7\times10^4\pm5.1\times10^3$	4	mL^{-1}	0.71	
Day 2	Total	$6.6\times10^4\pm6.1\times10^3$	4	$7.2\times10^4\pm1.0\times10^4$	4		0.39	
Day 6	Total	$1.5\times10^5\pm2.4\times10^4$	4	$1.3\times10^5\pm3.9\times10^4$	4		0.50	
Day 0	Noninfected	$3.5\times10^4\pm1.4\times10^3$	4	$3.5\times10^4\pm4.4\times10^3$	4		0.99	
Day 2	Noninfected	$6.5\times10^4\pm6.0\times10^3$	4	$6.7\times10^4\pm1.1\times10^4$	4		0.71	
Day 6	Noninfected	$1.3\times10^5\pm1.9\times10^4$	4	$8.7\times10^4\pm3.1\times10^4$	4		0.06	
Day 0	Infected	-	4	$2.9\times10^2\pm5.0\times10^2$	4		-	
Day 2	Infected	-	4	$1.6\times10^3\pm9.0\times10^2$	4		-	
Day 6	Infected	-	4	$2.5\times10^4\pm7.7\times10^3$	4		-	
Day 0	Postinfected	-	4	$5.3\times10^2\pm1.0\times10^3$	4		-	
Day 2	Postinfected	-	4	$9.7\times10^2\pm3.4\times10^2$	4		-	
Day 6	Postinfected	-	4	$1.0\times10^4\pm2.2\times10^3$	4		-	
Day 0	Decaying	$5.9\times10^2\pm3.8\times10^2$	4	$8.5\times10^2\pm2.0\times10^2$	4		0.29	
Day 2	Decaying	$1.5\times10^3\pm7.2\times10^2$	4	$2.2\times10^3\pm8.0\times10^2$	4		0.20	
Day 6	Decaying	$1.6\times10^4\pm6.0\times10^3$	4	$7.4\times10^3\pm2.0\times10^3$	4		0.06	
**Bacterial abundances**								
Free-living bacteria								
Day 0	-	$1.9\times10^6\pm7.0\times10^5$	75	$1.9\times10^6\pm6.7\times10^5$	82	mL^{-1}	0.12	
Day 2	-	$2.9\times10^6\pm1.6\times10^6$	80	$3.0\times10^6\pm1.0\times10^6$	80		0.84	
Day 6	-	$9.3\times10^6\pm3.3\times10^6$	80	$9.4\times10^6\pm3.2\times10^6$	76		0.95	
Diatom-associated bacteria with:							Group (Non-I)	Group (Inf)
Day 6	Noninfected diatom	11.1±9.7	276	9.2±7.6	219	$diatom^{-1}$	e	e
	Noninfected diatom (inf. colony)	-	-	15.2±9.7	104		-	d
	Infected diatom	-	-	22.4±16.7	219		-	c
	Postinfected diatom	-	-	34.1±17.4	142		-	b
	Decaying diatom	44.7±20.6	248	38.7±19.2	238		a	b
Total bacterial abundances								
Day 6	Assoc. with noninfected diatoms	$1.5\times10^6\pm1.3\times10^6$	$	$9.4\times10^5\pm8.5\times10^5$	$	mL^{-1}	-	-
	Assoc. with infected diatoms	-	$	$5.5\times10^5\pm4.5\times10^5$	$		-	-
	Assoc. with postinfected diatoms	-	$	$3.6\times10^5\pm2.0\times10^5$	$		-	-
	Assoc. with decaying diatoms	$7.1\times10^5\pm4.2\times10^5$	$	$2.9\times10^5\pm1.6\times10^5$	$		-	-
	Free-living	$9.3\times10^6\pm3.3\times10^6$	80	$9.4\times10^6\pm3.2\times10^6$	76		-	-
**FISH-identified bacterial taxa**								
Alphaproteobacteria (ALF968)								
Day 6	Assoc. with noninfected diatoms	44.9±16.5	51	38.1±14.5	40	%	a	ab
	Assoc. with noninfected diatoms (infected colony)	-	-	36.6±15.7	46		-	ab
	Assoc. with infected diatoms	-	-	31.2±13.9	58		-	b
	Assoc. with postinfected diatoms	-	-	32.7±14.6	43		-	b
	Assoc. with decaying diatoms	38.1±16.0	61	34.3±13.7	60		ab	b
	Free-living	25.6±6.0	60	22.1±5.5	60		0.00107**	
Gammaproteobacteria (BONE23A)								
Day 6	Assoc. with noninfected diatoms	29.7±14.1	59	25.3±17.6	49	%	c	c
	Assoc. with noninfected diatoms (inf. colony)	-	-	31.5±16.1	37		-	bc
	Assoc. with infected diatoms	-	-	43.9±22.2	62		-	ab
	Assoc. with postinfected diatoms	-	-	42.7±24.8	43		-	ab
	Assoc. with decaying diatoms	44.7±23.9	61	45.5±23.0	64		a	a
	Free-living	30.8±7.2	60	28.0±6.9	60		0.018*	
Bacteroidia (CF319a)								
Day 6	Assoc. with noninfected diatoms	27.7±15.5	45	30.9±18.1	42	%	a	a
	Assoc. with noninfected diatoms (inf. colony)	-	-	29.7±13.6	20		-	a
	Assoc. with infected diatoms	-	-	32.1±15.7	37		-	a

Assoc. with postinfected diatoms	-	-	36.4±12.8	16	-	a
Assoc. with decaying diatoms	34.1±15.1	56	35.6±13.6	55	a	a
Free-living	33.3±16.6	61	33.2±11.8	60	0.27	

† N denotes the numbers of replicates (*N*=incubation flasks for diatom abundances, *N*=fields of view for free-living bacteria, *N*=number of cells for *Asterionella*-associated bacteria)
‡ P-values are given for the two-sample comparisons (the statistical difference between data from the noninfected versus infected treatment. Letters a–f are given for multiple group comparisons (differences between multiple groups were tested separately for attached bacteria, free-living Alphaproteobacteria, free-living Gammaproteobacteria, and free-living Bacteroidia). If two groups share at least one letter, their data were not significantly different (Kruskal–Wallis test, $p>0.05$).
$ SD were calculated as propagating errors

SI Table S2. Characteristics of the noninfected and infected cultures on day 0, 2, and 6 after the chytrid inoculation.

Parameter	Time point	Noninfected treatment	Infected treatment	Unit	N†	P-value‡
Raw fluorescence	Day 0	21061±257	21659±798	relative fluorescence units (RFU)	4	0.23
	2	33919±1436	34236±910		4	0.72
	6	68535±3783	63482±3286		4	0.09
Chlorophyll a	0	32±2	33±2	ng mL^{-1}	4	0.42
	2	52±1	52±2		4	0.89
	6	89±3	85±1		4	0.07
Phaeopigments	0	1.5±0.3	2.3±1.5	ng mL^{-1}	4	0.36
	2	3.1±1.6	3.4±1.4		4	0.78
	**6**	**6.3±0.5**	**15.1±1.6**		**4**	**0.0008*** **
Particulate organic carbon (POC)	0	167.2±1.6	170.3±1.6	nmol mL^{-1}	4	0.11
	2	277.2±5.4	275.3±2.6		4	0.58
	6	616.8±26.5	595.5±28.7		4	0.13
Particulate organic nitrogen (PON)	0	25.3±0.5	25.6±0.4	nmol mL^{-1}	4	0.68
	2	40.7±0.8	40.9±0.3		4	0.79
	6	88.6±5.2	84.3±5.8		4	0.11
POC:PON ratio	0	6.6±0.1	6.7±0.1	mol : mol	4	0.36
	2	6.8±0.1	6.7±0.0		4	0.50
	6	7.0±0.1	7.1±0.2		4	0.16
Nitrate concentration (0.45 µm filtered)	0	417±11	412±5	nmol mL^{-1}	4	0.45
	2	409±3	411±6		4	0.44
	6	355±4	345±9		4	0.10
Soluble reactive phosphorus (SRP, 0.45 µm filtered)	0	38.9±0.6	38.3±0.3	nmol mL^{-1}	4	0.14
	2	37.3±0.3	37.0±0.3		4	0.21
	6	32.8±0.3	33.3±1.0		4	0.45
Silica (dissolved, 0.45 µm filtered)	0	1.56±0.14	1.53±0.01	nmol mL^{-1}	4	0.67
	2	1.21±0.04	1.21±0.02		4	0.83
	**6**	**0.20±0.02**	**0.23±0.01**		**4**	**0.03***
Dissolved organic carbon (DOC, (0.45 µm filtered)	0	66±1	69±5	nmol mL^{-1}	4	0.50
	2	102±4	115±4		4	0.07
	6	138±6	135±4		4	0.62
^{13}C-DOC	0	0.01±0.00	0.02±0.02	^{13}C-atom % excess	3	0.066
	**2**	**2.64±0.25**	**3.63±0.48**		**3**	**0.008***
	**6**	**3.96±0.36**	**4.89±0.03**		**3**	**0.0001****
DOC production rate	6	20.3±1.0	19.6±0.4	nmol C d^{-1} mL^{-1}	3	0.27

† N denotes the number of replicates (incubation flasks)

‡ P-values indicate statistical differences between noninfected vs. infected treatment

SI Table S3. Cell-specific ^{13}C and ^{15}N atom% excess and N-based growth rates in various cell types.

	^{13}C atom% excess (APE)				^{15}N atom% excess (APE)				N-based growth (d^{-1})		*N*	*N*
	Non-infected	Sig.	Infected	Sig.	Non-infected	Sig.	Infected	Sig.	Non-infected	Infected	Non-infected	Infected
**Diatom *Asterionella* and fungal cells (day 2)**												
Infected diatom	-	-	9.3±3.3	a	-	-	0.5±0.3	a	-	0.14±0.07	-	9
Chytrid sporangium	-	-	11.6±2.0	a	-	-	0.7±0.2	a	-	0.17±0.05	-	21
**Diatom *Asterionella* and fungal cells (day 6)**												
Noninfected diatom	6.8±1.0	d	7.2±1.1	c	1.8±0.2	a	1.7±0.2	ab	0.23±0.05	0.22±0.05	72	112
Noninfected diatom in infected colony	-	-	7.6±1.2	bc	-	-	1.6±0.2	c	-	0.19±0.05	-	120
Infected diatom	-	-	8.2±1.2	ab	-	-	1.6±0.2	c	-	0.19±0.05	-	49
Chytrid sporangium	-	-	8.4±1.3	a	-	-	1.7±0.2	bc	-	0.20±0.04	-	73
Chytrid zoospores	-	-	7.8±1.1	abc	-	-	1.7±0.2	abc	-	0.20±0.04	-	25
**Bacteria associated with (day 6)**												
Noninfected *Asterionella*	6.7±2.7	ab	6.5±3.3	b	1.5±0.2	ab	1.6±0.2	ab	0.18±0.03	0.18±0.05	87	45
Noninfected *Asterionella* in infected colony	-	-	8.4±2.2	a	-	-	1.6±0.2	a	-	0.19±0.04	-	50
Infected *Asterionella*	-	-	5.5±3.6	bc	-	-	1.4±0.2	c	-	0.15±0.04	-	224
Postinfected *Asterionella*	-	-	5.9±3.9	b	-	-	1.2±0.3	d	-	0.12±0.04	-	229
Decaying *Asterionella*	6.5±3.2	b	8.0±4.0	a	1.5±0.2	b	1.5±0.3	ab	0.17±0.04	0.17±0.04	384	180
Chytrid sporangia	-	-	2.9±0.6	c	-	-	1.5±0.2	bc	-	0.17±0.03	-	18
**Free-living bacteria (day 6)**												
DAPI-stained	2.2±0.8		2.3±1.1	(0.38)	1.3±0.2		1.2±0.3	(<0.0001)	0.14±0.04	0.12±0.04	194	344
ALF968-hybridised	1.8±0.5	d	2.0±0.6	cd (038)	1.1±0.2	b	1.0±0.2	c (0.0003)	0.11±0.02	0.09±0.02	56	60
BONE23A-hybridised	2.6±1.0	a	2.5±1.2	ab (0.69)	1.5±0.2	a	1.5±0.2	a (0.02)	0.18±0.04	0.16±0.04	73	79
CF319a-hybridised	2.2±0.6	bc	2.1±0.5	c (0.28)	1.2±0.2	b	1.1±0.2	b (0.02)	0.12±0.03	0.11±0.03	72	89

Significant differences between multiple group comparisons are shown as letters (Kruskal–Wallis test, run separately for each parameter in each of the four cell groups). Groups whose data distribution was statistically different do not share any letter ($p<0.05$). For FISH-identified free-living bacteria, an additional two-sample comparison was run to report exact *p*-values (noninfected vs. infected, Mann–Whitney test). N-based growth derived from single-cell ^{15}N-nitrate assimilation/transfer. N denotes the number of analyzed cells., Sig.– Significance (listing statistically significant differences).

SI Table S4. Taxonomic classification of the bacterial taxa (inferred from 16S rRNA gene amplicon sequencing, see also SI Appendix, Fig. S2).

Phylum	Class	Order	Family	Genus
Bacteroidota	Bacteroidia	Cytophagales	Spirosomaceae	*Lacihabitans*
Bacteroidota	Bacteroidia	Flavobacteriales	Flavobacteriaceae	*Flavobacterium*
Bacteroidota	Bacteroidia	Sphingobacteriales	env.OPS_17	*NA*
Bacteroidota	Bacteroidia	Chitinophagales	Chitinophagaceae	*Sediminibacterium*
Proteobacteria	Alphaproteobacteria	Caulobacterales	Caulobacteraceae	*Caulobacter*
Proteobacteria	Alphaproteobacteria	Caulobacterales	Caulobacteraceae	*Brevundimonas*
Proteobacteria	Alphaproteobacteria	Caulobacterales	Hyphomonadaceae	*Hirschia*
Proteobacteria	Alphaproteobacteria	Rhizobiales	Rhizobiaceae	*Allorhizobium-Neorhizobium-Pararhizobium-Rhizobium*
Proteobacteria	Alphaproteobacteria	Sphingomonadales	Sphingomonadaceae	*Novosphingobium*
Proteobacteria	Alphaproteobacteria	Sphingomonadales	Sphingomonadaceae	*Sandarakinorhabdus*
Proteobacteria	Gammaproteobacteria	Burkholderiales	Comamonadaceae	*Rhizobacter*
Proteobacteria	Gammaproteobacteria	Burkholderiales	Comamonadaceae	*Rhodoferax*
Proteobacteria	Gammaproteobacteria	Burkholderiales	Comamonadaceae	*Limnohabitans*
Proteobacteria	Gammaproteobacteria	Burkholderiales	Comamonadaceae	*Caenimonas*
Proteobacteria	Gammaproteobacteria	Burkholderiales	Comamonadaceae	*Polaromonas*
Proteobacteria	Gammaproteobacteria	Burkholderiales	Comamonadaceae	*NA*
Proteobacteria	Gammaproteobacteria	Burkholderiales	Oxalobacteraceae	*NA*
Proteobacteria	Gammaproteobacteria	Burkholderiales	Rhodocyclaceae	*Methyloversatilis*
Proteobacteria	Gammaproteobacteria	Gammaproteobacteria_Incertae_Sedis	Unknown_Family	*Acidibacter*
Proteobacteria	Gammaproteobacteria	Salinisphaerales	Solimonadaceae	*Nevskia*
**Others**				
Bacteroidota	Bacteroidia	Flavobacteriales	Flavobacteriaceae	*NA*
Proteobacteria	Alphaproteobacteria	Caulobacterales	Hyphomonadaceae	*UKL13-1*
Proteobacteria	Alphaproteobacteria	Reyranellales	Reyranellaceae	*Reyranella*
Proteobacteria	Alphaproteobacteria	Rhizobiales	Devosiaceae	*Devosia*
Proteobacteria	Alphaproteobacteria	Rhizobiales	Rhizobiales_Incertae_Sedis	*Phreatobacter*
Proteobacteria	Alphaproteobacteria	Rhizobiales	Xanthobacteraceae	*Tardiphaga*
Proteobacteria	Alphaproteobacteria	Rhodobacterales	Rhodobacteraceae	*Gemmobacter*
Proteobacteria	Alphaproteobacteria	Rhodobacterales	Rhodobacteraceae	*Pseudorhodobacter*
Proteobacteria	Alphaproteobacteria	Sphingomonadales	Sphingomonadaceae	*Sphingopyxis*
Proteobacteria	Gammaproteobacteria	Burkholderiales	Burkholderiaceae	*Limnobacter*
Proteobacteria	Gammaproteobacteria	Burkholderiales	Nitrosomonadaceae	*DSSD61*
Proteobacteria	Gammaproteobacteria	Salinisphaerales	Solimonadaceae	*Hydrocarboniphaga*
Proteobacteria	Gammaproteobacteria	Salinisphaerales	Solimonadaceae	*NA*

Others included taxa that comprised less than 2% of sequence reads in any of the samples

NA – not assigned

SI Table S5. Bacterial taxa with differential abundances in the noninfected versus infected culture on day 6 after the chytrid inoculation.

Class	Family	Genus	Normalized ASV counts	log2Fold Change Mean±SE	p-value	p-value (adjust)*
**DNA - Free-living bacteria**						
Bacteroidia	Spirosomaceae	*Lacihabitans*	465	0.57±0.18	1.18E-03	3.03E-02
Bacteroidia	Sphingobacteriales	env.OPS_17	184	0.97±0.22	1.28E-05	4.94E-04
α-proteobacteria	Caulobacteraceae	*Brevundimonas*	93	-4.22±0.33	3.13E-37	2.41E-35
**RNA - Free-living bacteria**						
Bacteroidia	Flavobacteriaceae	*Flavobacterium*	4046	1.58±0.32	7.35E-07	2.47E-05
Bacteroidia	Flavobacteriaceae	*Flavobacterium*†	144	1.53±0.35	1.04E-05	2.62E-04
α-proteobacteria	Caulobacteraceae	*Caulobacter*	154	2.26±0.42	5.36E-08	2.71E-06
α-proteobacteria	Caulobacteraceae	*Caulobacter*†	48	1.99±0.49	5.63E-05	1.14E-03
α-proteobacteria	Caulobacteraceae	*Caulobacter*†	5	3.21±1.01	1.46E-03	2.11E-02
α-proteobacteria	Caulobacteraceae	*Brevundimonas*	71	-3.84±0.54	7.44E-13	7.51E-11
**DNA - Diatom-associated bacteria**						
Bacteroidia	Spirosomaceae	*Lacihabitans*	233	0.59±0.18	1.14E-03	1.77E-02
α-proteobacteria	Caulobacteraceae	*Caulobacter*	73	0.97±0.24	7.32E-05	1.51E-03
α-proteobacteria	Caulobacteraceae	*Brevundimonas*	170	-5.67±0.29	3.11E-84	1.93E-82
α-proteobacteria	Caulobacteraceae	*Brevundimonas*†	5	-4.81±1.12	1.63E-05	5.05E-04
**RNA - Diatom-associated bacteria**						
α-proteobacteria	Caulobacteraceae	*Caulobacter*	256	1.85±0.50	2.00E-04	5.12E-03
α-proteobacteria	Caulobacteraceae	*Caulobacter*†	57	2.15±0.45	1.81E-06	6.95E-05
α-proteobacteria	Caulobacteraceae	*Caulobacter*†	8	2.06±0.70	2.99E-03	3.30E-02
α-proteobacteria	Caulobacteraceae	*Brevundimonas*	122	-6.23±0.71	2.52E-18	1.94E-16
α-proteobacteria	Caulobacteraceae	*Brevundimonas*†	3	-5.01±1.64	2.28E-03	3.30E-02

Tests were run on counts of individual ASVs. The normalized ASV counts of the listed taxa decreased or increased in the infected compared to the noninfected culture (indicated by negative or positive log2-fold changes, respectively, significance level = 0.01).
* Benjamini-Hochberg (BH) adjusted *p*-value
† listed multiple times since the corresponding ASVs were assigned to two different taxonomic units

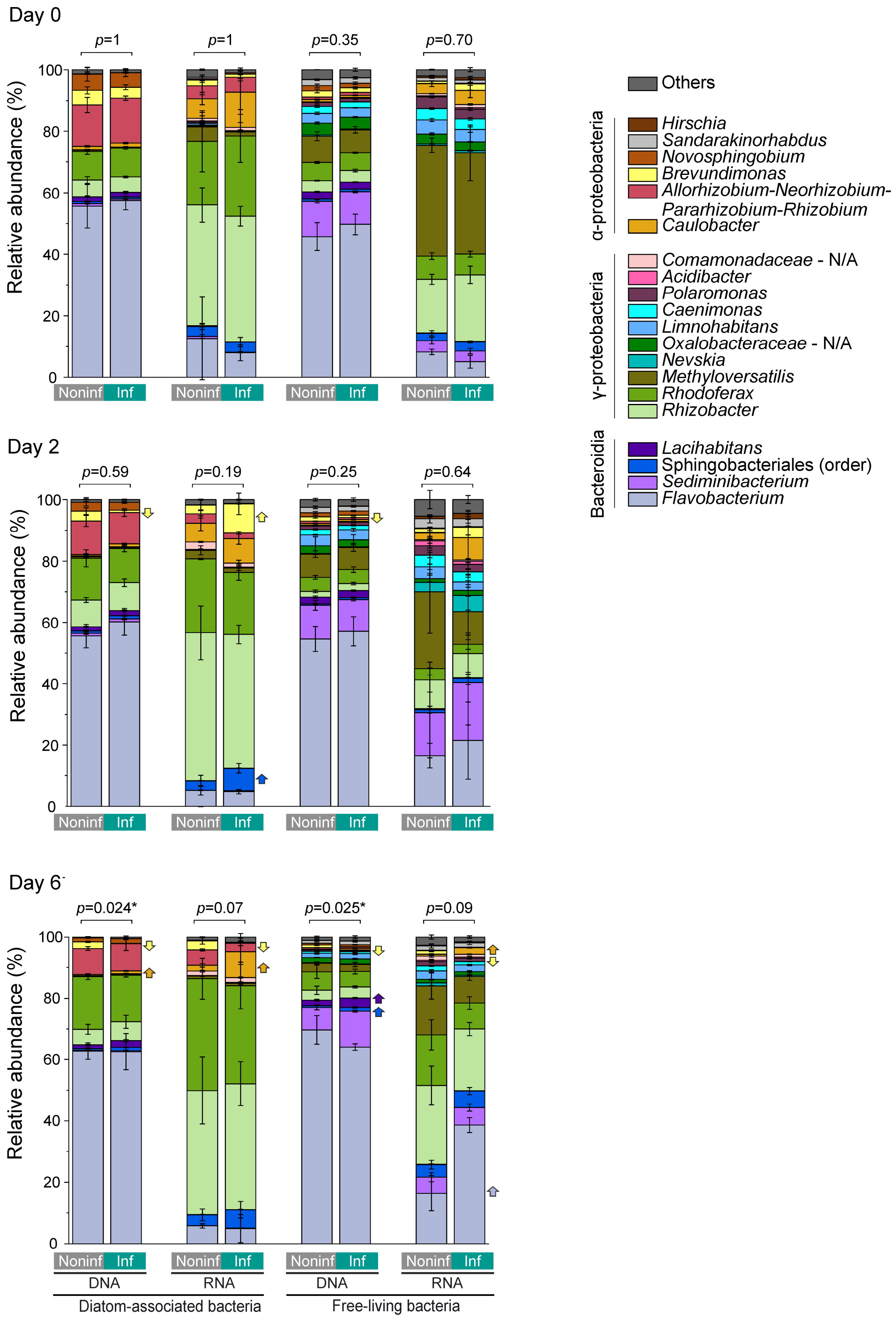

SI Figure S2. Bacterial community composition (16S rRNA gene-based, *i.e.*, DNA) and potential activity (16S rRNA-based, *i.e.*, RNA), shown as the relative abundance of ASV counts. Genus taxonomy is given, except for the order Sphingobacteriales (family *env.OPS17*), and the families *Comamonadaceae* and *Oxalobacteraceae*,

which did not have assigned genera (N/A). Statistical differences between the overall ASV composition in the noninfected vs. chytrid-infected treatment are indicated as *p*-values (derived from permutational ANOVA comparing weighted UniFrac distance metrics, asterisks indicate statistically significant differences, $p<0.05$). Arrows mark taxa with higher or lower ASV counts in the noninfected vs. chytrid-infected treatment ($p<0.01$). Taxa details are listed in SI Appendix, Table S4 and S5. Data are shown for day 0, 2, and 6 after chytrid inoculation.

Materials and Methods

SI Table S6. Sample overview.

Parameter	Method/ instrument	Volume (mL)	Preservation, storage	N*	Sub-replicates	Time points analyzed (day)	Comment
**Pigments, nutrients, and bulk isotope ratios**							
Raw fluorescence	Fluorometry	1.5	analyzed immediately	4		0, 2, 6	
Chlorophyll a/ phaeopigments	Fluorometry	5	90% acetone, -20°C	4		0, 2, 6	
Nitrate	FIA†/ spectrometry	1	GF75-filtered, -20°C	4		0, 2, 6	
SRP – soluble reactive phosphorous	FIA†/ spectrometry	1	GF75-filtered, -20°C	4		0, 2, 6	
Silica (dissolved)	FIA†/ spectrometry	7.5	GF75-filtered, -20°C	4		0, 2, 6	
DOC (dissolved organic C)	TOC‡ analyzer	5	GF75-filtered, -20°C	4		0, 2, 6	
^{13}C-DOC	IRMS	30	GF75-filtered, $ZnCl_2$, -20°C	3		0, 2, 6	Filtrate from POC / ^{13}C-POC, PON / ^{15}N-PON samples
^{13}C-DIC (dissolved inorganic C)	IRMS	12	GF75-filtered, $ZnCl_2$, RT	3		0, 2, 6	Filtrate from POC / ^{13}C-POC, PON / ^{15}N-PON samples
POC / ^{13}C-POC, PON / ^{15}N-PON	EA-IRMS	90	50°C drying, RT	3		0, 2, 6	
^{15}N-nitrate	IRMS	20	0.1 µm-filtered, -20°C	3		0, 2, 6	Filtrate from POC / ^{13}C-POC, PON / ^{15}N-PON samples
**Cell abundances**							
Diatoms/ infection prevalence	BF§ / fluorescence microscopy, WGA staining	2	Lugol, 4°C	4		0, 2, 6	
Zoospores	BF§ microscopy	2	Lugol, 4°C	4		0, 2, 6	
Bacteria (DAPI-stained)	Fluorescence microcopy, DAPI staining	0.5–1.0	PFA¶, -20°C	4	Free-living: 18–21 counting grids / replicate, Diatom-associated: 104–276 cells / diatom cell type	0, 2, 6	
FISH-identified bacteria (relative abundance)	CARD-FISH	0.5–1.0	PFA¶, -20°C	3	Free-living: 20 counting grids / replicate, Diatom-associated: 16–64 cells / diatom cell type and FISH probe	6	
**Nucleic acids**							
Bacterial community composition/ potential activity	16S rRNA gene/ 16S rRNA amplicon sequencing	20	Liquid N_2, -80°C	2–4		0, 2, 6	<4 replicates if ASV counts <500 reads, Size fractionation of diatom-associated bacteria (5.0 µm filter) and free-living bacteria (0.2 µm filter)
**Single-cell isotope ratios**							
Single-cell ^{13}C/^{12}C and ^{15}N/^{14}N ratio	IMS1280 / nanoSIMS - diatoms/sporangia	1.5–3.0	PFA¶, -20°C	3	9–120 cells / cell type	2, 6	
	NanoSIMS - zoospores	10–12			25 zoospores	6	
	NanoSIMS - cell-associated bacteria	1.5–3.0			18–384 bacteria / cell type	6	
	NanoSIMS - free-living bacteria	0.5–1.0			194/344 bacteria	6	
	FISH / NanoSIMS - free-living bacteria	0.5–1.0			56–89 bacteria / FISH probe	6	

* N - replicates represent Erlenmeyer flasks, in which the model system was grown (3x flasks with isotope amendment, 1x control without isotope amendments for each noninfected/infected treatment)
† FIA - flow injection analysis
‡ TOC - total organic carbon
§ BF - bright field
¶ PFA - paraformaldehyde

Characterization of the co-cultures

Raw fluorescence and pigments

1.5 mL were filled into a glass cuvette and analyzed immediately on a fluorometer (Trilogy Turner design, USA, Chl *a in vivo* Blue module). For pigment analyzes, 5 mL were transferred into 15 mL Falcon tubes and centrifuged at 3000 rpm for 20 min at 16°C. The supernatant was removed, the pellet extracted in 90% acetone, and frozen at -20°C until analysis on a fluorometer (436/680 nm, Hitachi Fluorescence Spectrometer F-7000). Chlorophyll *a* concentrations were calculated and corrected for phaeopigments, measured after acidification (1). Chlorophyll *a* was used as a standard (*Anacystis nidulans,* Sigma C6144, precision ±1%) and CHU-10 medium as blank.

Bulk isotope and nutrient analyses

For ^{13}C-dissolved inorganic C (DIC) analyses, 12 mL were filtered through GF/75 (Advantec, GF075.025, pore size 0.3 µm), filled headspace-free into Exetainer® vials and poisoned with $ZnCl_2$ (final 0.5% w/v). The ^{13}C-enrichment in the DIC pool was determined after chemical conversion of DIC to CO_2 with 85% phosphoric acid and subsequent trace gas isotope-ratio mass spectrometer (IRMS) analyses (precision ±0.1‰). Samples for ^{13}C-DOC were taken by filtering 30 mL through GF/75 into 50 mL Falcon tubes, adding $ZnCl_2$ (0.5% w/v), and storing at -20°C. DOC was chemically converted to CO_2 with sodium persulfate and analyzed on a TOC analyzer interfaced to an IRMS (precision ±0.4‰). For ^{15}N-nitrate analyses, 20 mL were filtered sequentially through GF/75 and 0.1 µm into polyethylene vials and stored at -20°C. The ^{15}N-enrichment in the nitrate pool was measured after converting nitrate to N_2O via the bacterial denitrification assay (2) and trace gas IRMS analysis (precision

±0.4‰). Samples for ^{13}C particulate organic carbon (POC) and ^{15}N particulate organic nitrogen (PON) were taken by filtering 90 mL onto pre-combusted GF/75 filters. Filters were dried at 50°C, fumed over HCl, pelletized into tin cups, and analyzed using elemental analyzer IRMS (EA-IRMS, precision ±0.2‰ for ^{13}C and ±0.3‰ for ^{15}N). Vienna PeeDee Belemnite served as C standard and air as N standard. Analyses of ^{13}C-POC/^{15}N-PON, ^{13}C-DIC, ^{13}C-DOC, and ^{15}N-nitrate were conducted at the UC Davis Stable Isotope Facility, California, USA.

Concentrations of nitrate, soluble reactive phosphorus, and dissolved silica were determined from 0.45 µm-filtered water by flow injection analysis (FIA) and spectrometric detection (ISO-13395-D28, ISO/DIS-15681-2, and DIN-38405-D21, respectively). DOC concentrations were analyzed from GF/75-filtered water with a TOC-V CPH (Shimadzu, Kyoto, Japan) using combustion catalytic oxidation. DOC production rates were defined as the sum of net DOC release and assimilation rates, calculated as

$$\text{Net DOC release rate} = \frac{\text{DOC concentration}_{t2} - \text{DOC concentration}_{t0}}{t_2 - t_0} \qquad \text{(Eq. 1)}$$

$$\text{Net DOC assimilation rate} = \frac{\text{Bacterial C biomass}_{t2} - \text{Bacterial C biomass}_{t0}}{t_2 - t_0} \qquad \text{(Eq. 2)}$$

In Eq. 2, we assumed that the increase in bacterial C biomass derived entirely from DOC assimilation.

Abundances of diatoms, zoospores, and sporangia

Two replicates of 2 mL were transferred into Eppendorf tubes, preserved with Lugol (5 vol%), and stored at 4°C in darkness. One of the replicates was counted within the

next day, to enumerate the abundance of free-living zoospores. The additional replicate was utilized later to count diatoms and attached sporangia. Samples were de-stained from Lugol with $Na_2S_2O_3$ and subsequently stained with Wheat Germ Agglutinin (WGA conjugated to Alexa Fluor™ 488, 2.5 µg mL^{-1}) to visualize the chitinous cell walls of sporangia (3). *Asterionella* cells were differentiated and counted as (i) noninfected, (ii) infected, (iii) postinfected, and (iv) decaying cells in Utermoehl plankton chambers (Hydrobios) under an inverted fluorescence microscope (Nikon TiE Inverted Microscope, 400x magnification).

Bacterial community composition and abundances

16S rRNA gene (DNA) and 16S rRNA (RNA/cDNA) extraction, amplification, and sequencing

Bacterial communities were size-fractionated via filtration. In detail, bacteria associated with large diatoms (>5 µm) were collected onto 5.0 µm polycarbonate filters (PC, Whatman) by filtering 20 mL. Free-living bacteria were thereafter collected onto 0.2 µm PC filters from the 5.0 µm-filtrate (*i.e.*, the water that passed through the 5.0 µm filters). The low-pressure during filtration was low with 50−100 mbar to minimize any detachment of bacteria from the diatoms. Filters were transferred into cryovials, flash-frozen in liquid N_2, and stored at -80°C. DNA and RNA were extracted after (4, 5). To each filter, we added zirconia-silica beads (0.1–1.0 mm), 650 µL 2% CTAB-buffer, 65 µL 10% sodium dodecyl sulfate, 65 µL 10% lauroyl sarcosine, and 10.6 µL Proteinase K (20 mg mL^{-1}). After 1 h at 60°C, 650 µL phenol:chloroform:isoamyl alcohol (25:24:1, v/v) were added and the solution exposed to a bead-beating step (40 s, speed 6 m s^{-1}, FastPrep-24™, MP Biomedicals) followed by centrifugation (10 min, 14,000 g, 4°C). The aqueous phase

was mixed with chloroform:isoamyl alcohol (24:1) and again centrifuged. Nucleic acids were extracted in 30% PEG6000/1.6 M NaCl plus 1.2 µl GenElute™-LPA (linear polyacrylamide) for 1 h at 4°C (4). After centrifugation (1 h, 17,000 g, 4°C), the pellets were washed twice with iced 70% ethanol, resuspended in nuclease-free water (30 min, 37°C), and stored at -80°C. RNA elutions were treated with Turbo DNAse (ThermoFisher, AM2238) to remove co-extracted DNA and subsequently converted to cDNA using an Array Script Reverse Transcriptase (AM2048). Successful DNA removal prior to cDNA synthesis was confirmed by the lack of a PCR amplification product, as visualized via gel electrophoresis, and also validated later by low sequence reads after amplicon sequencing (0–15 ASV counts per sample).

The 16S rRNA-encoding DNA and cDNA were amplified using the primer set 515F/926R (6) following (7). As modifications, DNA and cDNA were amplified using a Takara Master Mix (Hot Start Taq Polymerase, RR006A, TaKaRa, Japan). Each PCR program included 180 s at 95°C, 30 cycles of 45 s at 95°C, 45 s at 50°c, and 90 s at 68°C, and finally 300 s at 68°C. As an internal positive control, we used a stacked mock community (6), to reveal possible biases throughout the workflow and analysis. As negative controls, we used blank samples with no DNA added during the DNA/cDNA extraction, and blanks with no DNA/cDNA added during amplification. Duplicate samples (*i.e.*, technical replicates) were created by adding the same DNA/cDNA extract for amplification. All samples were randomized. Positive, negative, and duplicate controls were inserted every 15th sample. Barcodes were added to the adaptor sequences on the 515F/926R primer set using a Platinum Taq Polymerase (ThermoFisher, 10966). The PCR program included 180 s at 95°C, 8

cycles of 30 s at 95°C, 30 s at 55°c, and 30 s at 72°C, and finally 300 s at 72°C. Amplified and barcoded PCR products were purified using AGENCOURT® AMPURE® XP beads (A63881, Beckman Coulter, CA, USA) and a magnetic separator, and finally pooled (2 ng DNA μL^{-1} for each sample). Illumina Sequencing (MiSeq PE250) was performed at the UC Davis Genome Center (DNA Technologies and Expression Analysis Core Facility).

Amplicon sequencing data analysis

Primers were removed from the sequences with cutadapt (2.4). Truncation, quality filtering, dereplication, pooling, and chimera removal were done with dada2 (1.12.1) (8) in R (v. 3.6.1) (9). For quality filtering, a maximum of two erroneous bases was allowed for forward and reverse reads, and ten base pairs were used as an overlap when merging forward and reverse reads. For assigning taxonomy to each amplicon sequence variant (ASV), the SILVA 138 SSU database was used as a reference. Likely contaminants were detected and removed using decontam (1.4.0). Relative abundances of ASV counts were plotted after normalizing for sample depth with DESeq2 (1.18.1), and after discarding sequences assigned to chloroplasts, mitochondria, eukaryotes, and archaea. Moreover, samples with <500 sequences (final range ~500–50,000) and ASVs with <20 counts in the entire data set were excluded. Differential abundance analyses of single taxa were done on normalized ASV counts with DESeq2. To indicate any changes in the total community structure, the variance between samples was tested via permutational ANOVA (adonis function, vegan 2.5.6) using a weighted UniFrac distance metric (10). The weighted UniFrac distance was calculated from ASV count data after variance stabilizing transformation and incorporating the phylogenetic distance based on a rooted tree

(UPGMA), which we constructed with phangorn 2.5.5. Data homogeneity within-group dispersion was validated with the betadisper function. Besides the weighted UniFrac distance, we also tested other metrics like Jensen-Shannon Divergence (JSD) and Bray–Curtis, whose data ordination explained similarly high proportions of variabilities (47–95%) on a two-dimensional PCoA. Moreover, statistical differences that derived from permutational ANOVA showed similar patterns for JSD, Bray-Curtis, and weighted UniFrac (except for the comparison of potential activity, *i.e.*, 16S rRNA, of free-living bacteria in noninfected versus chytrid-infected cultures on day 6, for which Bray–Curtis and JSD indicated a statistically significant difference (p=0.03), as opposed to weighted UniFrac (p=0.09).

Bacterial abundances and Catalyzed Reporter Deposition Fluorescence in situ Hybridization (CARD-FISH)

0.5–1 mL were preserved with paraformaldehyde (PFA, final concentration 1.5%) overnight, thereafter filtered onto PC filters (0.2 µm, 25 mm, Whatman), and stored at -20°C. Total bacterial abundances were determined on filters that were WGA-stained and mounted in Vectashield with DAPI (H-1200, Vector Laboratories, Burlingame, CA, USA). CARD-FISH was used to visualize and quantify the major bacterial taxa, as inferred from the amplicon sequencing analyses. The probes included ALF968 (for Alphaproteobacteria) (11), CF319a (for Bacteroidia) (12), and BONE23A (13) (specific to the gammaproteobacterial order Burkholderiales, which were formerly classified as Betaproteobacteria (14)). CARD-FISH was done only on cells sampled on day 6, whereas total bacterial abundances were determined on day 0, 2, and 6 after chytrid inoculation.

CARD-FISH was performed on filter sections cut from the same filters that were used for total bacterial counts following (7, 15) but using one separate filter section for each probe. Briefly, cells were permeabilized with Lysozyme (10 mg mL^{-1}, 1 h at 37°C). Lysozyme and endogenous peroxidases were inactivated with 0.01 M HCl. Hybridization was done with horseradish peroxidase-labeled oligonucleotide probes in 35% formamide (3 h at 46°C). Signal amplification was achieved with tyramides conjugated to Alexa Fluor™ 555 (ThermoFisher). Before counting, cells were mounted and counterstained in Vectashield with DAPI, to determine the percentage of hybridized cells relative to DAPI-stained bacteria.

Bacteria that were only DAPI-stained but had not undergone any FISH protocol were counted directly under the microscope, whereas FISH-treated cells were imaged as z-stacks (0.1 µm increments), to directly overlay DAPI-stained (DAPI-channel, 360/460 nm) and hybridized cells (DsRed-channel, 560/645 nm). For free-living bacteria, z-stack images were combined to one composite image with one focal plane using picolay (v.3, www.picolay.de) and further processed in ImageJ (v.1.51.p) (16). In ImageJ, each color image was converted into a binary (black/white) image. Fluorescent cells were counted automatically via particle analysis after setting a manual threshold to separate bright pixels (fluorescent cells) from dark pixels (background). Diatom-associated bacteria were often overlapping on z-stacked images, impeding the automatic counting. Thus, diatom-associated cells were counted manually on z-stacks.

Bacteria were grouped into those being associated with (i) noninfected, (ii) infected, (iii) postinfected, and (iv) decaying *Asterionella* cells, and into (vii) free-living bacteria

(Figure 1). Total bacterial abundances were counted before CARD-FISH, to account for any potential loss of bacteria from the filters during the CARD-FISH protocol. Yet, we could not detect such loss, and thus, we merged data from both countings (before and after CARD-FISH). For each group and replicate, we examined 16–276 *Asterionella* cells (for diatom-associated bacteria) and 60–82 counting grids (for free-living bacteria). The performance of CARD-FISH and image analyses were validated with the EUB338 probe (17), showing a hybridization efficiency of 101±8% (n=38 fields of view). Negative controls with nonEUB338 (complementary sequence to EUB338) (18) successfully excluded any non-specific binding.

Secondary Ion Mass Spectrometry (SIMS)

Sampling for SIMS analyses

Cells were preserved with PFA (1.5% final concentration) overnight at 4°C prior to filtration and stored at -20°C. To collect diatom-associated bacteria, we filtered 1.5–3.0 mL onto 5.0 µm isopore membrane filters (TMTP02500, Merck, Darmstadt, Germany), to reach sufficiently high diatom abundances and to exclude free-living bacteria. Zoospores were collected by pre-filtering 10–12 mL through 5.0 µm, to remove large diatoms, and collecting the filtrate onto 1.2 µm isopore membrane filters (RTTP02500). For free-living bacteria and some diatoms plus sporangia analyses, we used the same filters (0.5–1.0 mL onto 0.2 µm) as for the bacterial counting. Before IMS 1280 analyses, each filter section was imaged at 200x magnification under a scanning electron microscope (Benchtop SEM, JCM600, Tokyo, Japan), to be able to relocate noninfected and infected *Asterionella* colonies with the optical camera under the IMS 1280. For NanoSIMS analyses, cells were imaged at 1000x magnification under a fluorescence microscope. Additionally, we

took an overview image of the entire filter section (100x magnification), to facilitate the relocation of cells under the NanoSIMS.

IMS 1280 analyses

Filters were cut into 4×4 mm sections, glued onto adhesive carbon SEM tape on a glass slide (25 mm), and coated with a ca. 5 nanometer-thick gold layer before secondary-ion imaging using the IMS 1280, which followed procedures described in (19). Areas of interest were pre-sputtered with a primary cesium ion beam (Cs^+, 10 nA) to remove the gold coating and chitinous cell wall of sporangia or silica frustules of diatoms. The pre-sputter time was 90 sec for the parasitic sporangia and 360 sec for *Asterionella*. Data acquisition was performed on 90×90 µm rasters (256×256 px resolution) with an 80–100 pA Cs^+ beam, utilizing the synchronized secondary ion beam raster (dynamic transfer optical system, DTOS) to maintain high transmission and high mass resolution, and to facilitate reconstruction of the ion image by the instrument software. The secondary ion signals of $^{13}C^{14}N^-$, $^{12}C^{14}N^-$ and $^{12}C^{15}N^-$ were recorded by a low-noise (<0.005 counts per second) ion counting electron multiplier sequentially over 80 planes using a peak-switching routine with dwell times of 15, 76, and 31 µs px^{-1} $plane^{-1}$, respectively. A mass resolution of ca. 12,000 (M/ΔM) was used to resolve potential interference of $^{11}B^{16}O$ (mass 27.00422) from $^{13}C^{14}N$ (mass 27.00643), given that diatom frustules can include traces of boron (20), which may not have been entirely removed (or were partly re-implanted) by the pre-sputter.

NanoSIMS analyses

Filters were cut into sections (2–4 mm) and either WGA and DAPI stained or, for FISH–SIMS, first treated according to the CARD-FISH protocol and subsequently stained with WGA and DAPI. After cell imaging under the fluorescence microscope, the mounting agent and any remaining stain were washed off sequentially in sterile water, 50% ethanol, and 100% ethanol as described in (21). Thereafter, the filter sections were glued onto sticky carbon SEM tape and coated with gold, similar to IMS 1280 samples. The pre-sputter time was 90–180 sec (for bacteria and sporangia, ca. 40–50 pA at D1-3) or 20–30 min (for diatoms and sporangia, ca. 400–500 pA at D1-0). Data acquisition was conducted on 25×25 µm rasters (256×256 px resolution) with a 4–5 pA Cs^+ beam. Secondary ions were collected simultaneously into seven separate collectors set to the masses of the ion species $^{12}C^-$, $^{13}C^-$, $^{12}C_2^-$, $^{12}C^{13}C^-$, $^{14}N^{12}C^-$, $^{15}N^{12}C^-$, and $^{28}Si^-$ over 40–50 planes. The dwelling time was 1 ms px^{-1} $plane^{-1}$. The mass resolution was ca. 8,000–10,000 (M/ΔM) (as specified by the CAMECA software for $^{12}C^{14}N^-$).

SIMS image processing

Images were processed in WinImage II. On IMS 1280 images, ROIs were drawn automatically using the ROI extraction tool, including only areas with high CN^- counts (the threshold on each image was set to 0.35-times the maximum $^{12}C^{14}N^-$ count value). For NanoSIMS images, this tool was not applicable, since it failed to perfectly separate adjacent bacterial cells. Therefore, ROIs were drawn manually on the $^{12}C^{14}N^-$ ion images, based on the direct overlay with the corresponding microscope images. In diatoms and some sporangia, the CN biomass was often heterogeneously distributed (*e.g.*, see Figure 3C), leading to multiple ROIs per cell, of which we

averaged the obtained ratios for each cell. For each ROI, isotope ratios were averaged over 80 (IMS 1280) or 40–50 planes (NanoSIMS 50L). If the standard deviation (SD) of the average ratio was >25%, the ratios were checked manually on a depth profile, to find at least 20 consecutive planes for which SD was <25%. Similarly, we set the threshold of the Poisson distribution for each ROI to SD <5%. In total, 95% of all ROIs passed this quality check, while the remaining 5% were discarded. For each cell type sampled at day 6, we analyzed 18–384 cells, yielding representative mean isotope ratios with SE<5% (except for bacteria associated with noninfected *Asterionella* in the infected treatment, SE=8% for ^{13}C APE).

$^{13}C/^{12}C$ ratio (NanoSIMS 50L)

During NanoSIMS analyses, we simultaneously collected the masses of the ions $^{13}C^-$, $^{12}C^-$, $^{12}C^{13}C^-$, and $^{12}C_2^-$. Thus, we could calculate $^{13}C/^{12}C$ ratios as $^{13}C^-/^{12}C^-$, and $^{12}C^{13}C^-/^{12}C_2^-$ ×0.5. Both ion pairs resulted in ratios following a close to 1:1 ratio, before and after correction for instrumental mass fractionation (see below), with slopes of linear regression of 1.08 and 0.89, respectively (SI Appendix, Fig. S3A). A similar trendline equation has been reported for the ratio $^{13}C^{14}N^-/^{12}C^{14}N^-$ versus $^{13}C^-/^{12}C^-$ (y=1.13x + 1.13) (22), while others mentioned no difference in ^{13}C contents inferred from C_2^- signal intensities as opposed to C^- signal intensities (C^-) (23, 24). Nevertheless, we used the ion-pairing $^{12}C^{13}C^-/^{12}C_2^-$ over $^{13}C^-/^{12}C^-$ for calculating $^{13}C/^{12}C$ ratios due to three reasons (see below for one exception when using $^{13}C^-/^{12}C^-$ for calculations):

(1) The instrumental mass fractionation (IMF) was substantially lower for $^{12}C^{13}C/^{12}C_2$ ×0.5 ratios (0.5−2%) than for $^{13}C/^{12}C$ ratios (16–21%).

(2) During analyses, the E0S focusing voltage was optimized for $^{12}C^{14}N^-$. This optimization aligns well with the peak transmission of $^{12}C_2^-$ but less well for that of $^{12}C^-$. That is, on a scan of the secondary-ion beam focus voltage, the peak of $^{12}C^-$ transmission is commonly a few volts off as compared to the $^{12}C^{14}N^-$ peak. For instance, Pett-Ridge and Weber (25) show an offset of -25 V while we found an offset of -50 V. This offset leads to a non-optimal $^{12}C^-$ transmission, implying a non-optimal accuracy in $^{12}C^-$ ion counts (which may also, at least partly, explain the lower IMF for $^{12}C^{13}C^-/^{12}C_2^-$ than for $^{13}C^-/^{12}C^-$).

(3) The di-atomic ions $^{12}C_2^-$ and $^{13}C^{12}C^-$ showed a higher ionization yield compared to the mono-atomic $^{12}C^-$ and $^{13}C^-$ (SI Appendix, Fig. S3B), leading to a higher precision of $^{13}C/^{12}C$ ratios. This higher precision was reflected by lower SDs of ratios averaged over 40–50 planes and lower Poisson errors for single ROIs (SI Appendix, Fig. S3C and D).

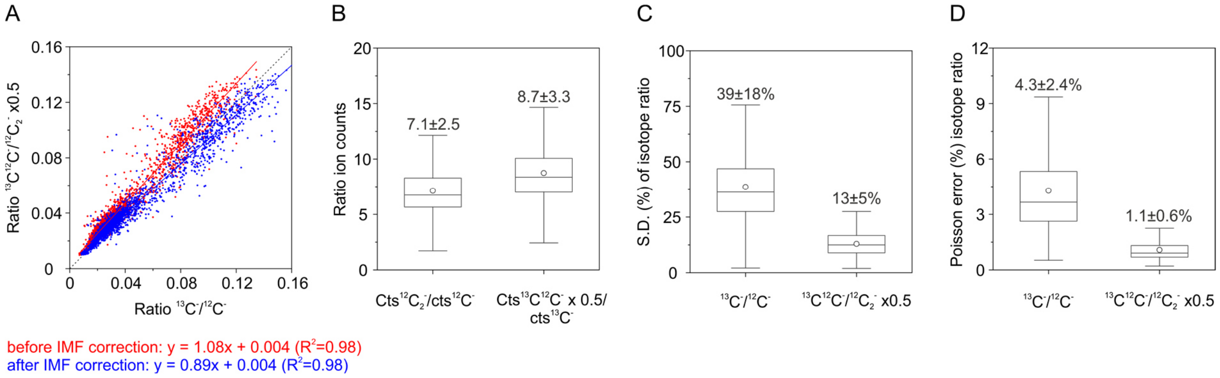

SI Figure S3. (A) Comparison of $^{13}C/^{12}C$ ratios (before and after IMF correction) calculated from the two ion pairings $^{13}C^-/^{12}C^-$ and $^{12}C^{13}C^-/^{12}C_2^-$, which were collected simultaneously during NanoSIMS analyses. The dotted line indicates a 1:1 ratio. **(B)** The number of ion counts (cts) was higher for the di-atomic C_2^- ions compared to the mono-atomic C^- ions, resulting in lower deviations and percent errors. **(C)** Standard deviations represent the deviation of ratios averaged over 40–50 planes. **(D)** Poisson errors represent a theoretical precision of the mean, assuming that the ion counts within each ROI are random variables with Poisson distribution. **(B–D)** Means±SDs are shown on top of the whiskers (each with *n*=2844).

Instrumental mass fractionation (IMF) and isotope dilution factor (DF)

Isotope ratios were corrected for IMF during NanoSIMS and IMS 1280 analyses, following (26). As a reference, we used the isotope ratios of control cells (without isotope amendment) analyzed via EA-IRMS (see above). IMF was ≤2% for NanoSIMS and IMS 1280 analyses, except for 35 cells of Gammaproteobacteria. Those cells were analyzed in a NanoSIMS run during which we only collected $^{13}C^-$ and $^{12}C^-$ ions (not $^{12}C^{13}C^-/^{12}C_2^-$) for $^{13}C/^{12}C$ ratio calculations. The corresponding IMF was 21% for $^{13}C/^{12}C$; however, after IMF correction, isotope ratios were not significantly different between those 35 cells and an additional 44 cells, which we analyzed in a run with IMF=0.5%. (p=0.92, Df=77). Thus, the mentioned 35 cells were included in the final data set.

We also corrected isotope ratios for isotope dilution due to chemical treatments during sample preparation (27, 28), following (28, 29). We determined approximate isotope dilution factors (DFs) and used them to estimate original isotope ratios. In detail, isotope ratios of *Asterionella* cells measured before (IMS 1280) and after DAPI-staining (NanoSIMS) showed no significant difference (p≥0.18). We propose that the dilution effect due to DAPI-staining was absent in *Asterionella* cells since DAPI-stained DNA was located in the nucleus, which covered a rather small area of the entire ROIs. In contrast, ROI areas of bacteria, sporangia, and zoospores were almost entirely stained with DAPI. Accordingly, isotope ratios of zoospores and sporangia were significantly higher before than after DAPI-staining, revealing DFs of 0.14 (for $^{13}C/^{12}C$) and 0.06 (for $^{15}N/^{14}N$). These fungi-derived DFs were approximately similar to previously reported bacteria- and archaea-derived DFs (0–0.08 for $^{13}C/^{12}C$

and 0–0.07 for $^{15}N/^{14}N$) (28). For consistency purposes, we applied the fungi-derived DFs also to DAPI-stained bacteria for which we did not conduct any SIMS analyses before DAPI-staining. The CARD-FISH protocol led to an additional isotope dilution for FISH-identified bacteria. We compared the mean isotope ratios of DAPI-stained bacteria (not subjected to CARD-FISH) with those of FISH-identified cells (subjected to CARD-FISH and positively hybridized, averaged for all three FISH-taxa). The resulting DFs were 0.01 ($^{13}C/^{12}C$) and 0.20 ($^{15}N/^{14}N$), leading to final DFs for FISH-identified cells of 0.15 ($^{13}C/^{12}C$) and 0.25 ($^{15}N/^{14}N$). Note that these DFs represent an approximation since they have been shown to vary for different microbial taxa (28). On average, we observed less isotope dilution from CARD-FISH than observed by Meyer, Fortney and Dekas (28), likely due to different analyzed taxa and/or differences in protocols (*e.g.,* fixative agent 1.5% PFA instead of 3% PFA or ethanol, permeabilization agent lysozyme instead of proteinase K, and formamide concentration 35% instead of 20%). The final DFs were in the lower range of previously reported DFs (28). Nonetheless, our results are largely insensitive to the magnitude of the DF applied since most measurements were made on cells that did not undergo FISH, and for those that did we present relative comparisons of ^{13}C and ^{15}N enrichments between cell groups rather than absolute values.

Calculating C transfer and cell-specific and population-specific growth rates

Isotope ratios were expressed as atom% excess (APE)

$$APE = \left[\frac{R_{cell}}{R_{cell}+1} - \frac{R_i}{R_i+1}\right] \times 100\% \qquad \text{(Eq. 3)}$$

where R_i is the initial cellular isotope ratio before incubations (derived from control cells, without isotope amendments) and R_{cell} is the isotope ratio after incubations.

The percent C transfer efficiency to each cell group and the DOC pool during the 6-day incubations was estimated as

$$\%\ \text{C transfer efficiency} = \frac{\text{Mean APE}_{\text{consumer cell or DOC pool}}}{\text{Mean APE}_{\text{source cell}}} \times 100\% \qquad \text{(Eq. 4)}$$

This transfer efficiency index represents an approximation for the various cell types, which grew based on different nutrition modes and lifestyles (*e.g.*, parasitic chytrids, epiphytic/mutualistic and saprophytic/opportunistic bacteria, and free-living and cell-associated cells). Uncertainties (± SD) in transfer efficiencies derived from combined uncertainties of each variable, following the laws of error propagation. Cell-specific N-based growth rates were calculated based on the fraction of newly incorporated N by single cells, relative to their initial N content (30)

$$K_A = \frac{R_{cell} - R_i}{(1 + R_i) \times [F_{source} \times (1 + R_{cell}) - R_{cell}]} \qquad \text{(Eq. 5)}$$

where F_{source} is the fraction of the heavy isotope in the substrate pool. K_A has also been described as Fx_{net} by (26, note the revision of the Fx_{net} equation in comparision to the book chapter from 2012). Cell-specific N-based growth rates μ (d^{-1}) were calculated as

$$\mu_{Cells} = \frac{\ln(1 + K_A)}{(t_2 - t_0)} \qquad \text{(Eq. 6)}$$

These growth rates refer to the N-based growth in single cells derived from ^{15}N-nitrate incorporation (directly or indirectly via transfer). For comparison, we also calculated growth rates for entire cell populations, based on our cell counts following (31)

$$\mu_{Population} = \frac{\ln(N_2) - \ln(N_0)}{(t_2 - t_0)} \qquad \text{(Eq. 7)}$$

where N_2 and N_0 are the cell abundances at *t2* and *t0*.

For ^{15}N-nitrate, F_{source} was 2.78±0.02 atom% (n=7). For ^{13}C-DIC, F_{source} decreased during the incubations (from 40.0±0.2 atom% to 2.7±0.4 atom% after 6 days) due to an open diffusive gas exchange between the culture medium and the atmosphere. As a result, cell-specific $^{13}C/^{12}C$ ratios partly exceeded the average substrate labeling, and hence, we did not calculate C-based growth rates (as done for N). Instead, we used mean $^{13}C/^{12}C$ ratios as a relative measure for the transfer of photosynthetically-derived C from *Asterionella* to the other cell types.

Statistical analyses

Statistical differences between two (non-paired) samples were calculated using the Mann–Whitney test for non-normally distributed data, the Welsh-test for normally distributed data with non-equal variance, and the *t*-test for normally distributed data with equal variance. Normal distribution was verified using the Shapiro-test and data variance with the F-test. Statistical differences between multiple groups were determined with the Tukey's HSD test (for normally distributed data populations) and the Kruskal–Wallis test (if normal distribution was rejected, with Bonferroni correction for *p*-value adjustment). Tests were run in R3.6.1 and OriginPro2020b.

Extrapolating the transfer of photosynthetically-derived C during chytrid infections in a natural plankton community

Cell abundances: Diatom and chytrid abundances were derived from (32) who followed the population dynamics of a natural *Asterionella* population during a chytrid epidemic in Lake Maarsseveen (Netherlands). Diatom abundances were reported to

have increased from ~100 to 400 cell mL^{-1}, with an infection prevalence of 2–54% over 50 days in February/March 2009. Gsell*, et al.* (32) did not distinguish between infected, postinfected, and decaying *Asterionella* cells but we assumed a ratio of 2:1 for infected to postinfected cells, and decaying cells were assumed to comprise 4% of all *Asterionella* cells, similar to our incubations. The abundance of sporangia was set equal to the abundance of infected *Asterionella,* thus accounting only for singular infections on individual diatom cells. The abundance of free-swimming zoospores was modeled as a function of infection prevalence and the number of post-/infected diatom cells, based on data from this study and (33). The obtained function was: $Zoospores\ (mL^{-1}) = 0.0824e^{0.0311 \times prevalence\ (\%)} \times post\text{-}/infected\ diatoms\ (mL^{-1})$.

Total abundances of cell-associated bacteria were calculated by multiplying the number of associated bacteria per *Asterionella* with the abundances of *Asterionella* with different health/infection status. The amount of DOC released from *Asterionella* was assumed to equal ~0.30-times the POC content of all *Asterionella* cells, as shown for our culture incubations.

Cell-specific C content: Based on our POC (EA-IRMS) and *Asterionella* abundance (microscopy) data, the C content of individual healthy *Asterionella* cells was 4.05 pmol C $cell^{-1}$ or 49 pg C $cell^{-1}$, in agreement with (33) who reported 46 pg C $cell^{-1}$. Cell-specific C contents of post-/infected and decaying diatoms, sporangia, zoospores, and bacteria were estimated from the areas of their ROIs on the $^{12}C^{14}N$ images derived from SIMS analyses (visualizing the areas of CN-biomass in individual cells). The cross-sectional area of each cell-specific ROI was used to calculate the equivalent spherical diameter ($ESD = 2 \times \sqrt{Area / \pi}$) and volume ($Volume = 4/3\,\pi \times (ESD/2)^3$). For elongated *Asterionella*, ROI volumes were calculated as $4/3\,\pi \times (ESD/2)^2 \times 0.75$, where 0.75 represented the minor axes of

ROIs. The resulting ROI volumes of infected, postinfected, and decaying *Asterionella* cells corresponded to 0.46, 0.15, and 0.09-times the ROI volume of noninfected *Asterionella* cells, respectively (see also SI Appendix, Table S7). To calculate the amount of photosynthetically-derived C for each cell type during the 50 days, the respective cell abundances and cell-specific C contents were multiplied.

SI Table S7. Dimensions of cell-specific ROIs on SIMS images (displaying CN-biomass) and estimated C contents.

**Cell type**	**Volume (ROI) µm^3**	**C content pmol C cell^{-1}**	**Number of cells**
Diatoms			
Noninfected	20.0±13.5	4.05±0.31*	305
Infected	9.2±7.9	1.86	49
Postinfected	3.0±5.0	0.62	5
Decaying	1.8±0.8	0.37	2
Fungal parasites (chytrids)			
Sporangia	20.3±21.0	2.19	151
Zoospores	1.2±0.9	0.15†	26
Bacteria			
Free-living and associated	0.23±0.20	0.005±0.003 ‡	2172

* calculated based on POC and *Asterionella* abundance data from the noninfected culture, *N*=3 (day 0, 2, and 5)
† assuming that 15 new zoospores developed in each sporangium (34)
‡ after (35)

References

1. E. J. Arar, G. B. Collins (1997) *In vitro* determination of chlorophyll and phaeophytin a in marine and freshwater algae by fluorescence. in *Method 445.0* (U.S. Environemental Protection Agency, Cinncinnati, Ohio).
2. D. M. Sigman *et al.*, A bacterial method for the nitrogen isotopic analysis of nitrate in seawater and freshwater. *Anal. Chem.* **73**, 4145-4153 (2001).
3. M. Meyberg, Selective staining of fungal hyphae in parasitic and symbiotic plant-fungus associations. *Histochemistry* **88**, 197-199 (1988).
4. O. Nercessian, E. Noyes, M. G. Kalyuzhnaya, M. E. Lidstrom, L. Chistoserdova, Bacterial populations active in metabolism of C1 compounds in the sediment of Lake Washington, a freshwater lake. *Appl. Environ. Microbiol.* **71**, 6885-6899 (2005).
5. P. Bossier *et al.*, An RFLP database for authentication of commercial cyst samples of the brine shrimp *Artemia* spp. (International Study on Artemia LXX). *Aquaculture* **231**, 93-112 (2004).
6. A. E. Parada, D. M. Needham, J. A. Fuhrman, Every base matters: Assessing small subunit rRNA primers for marine microbiomes with mock communities, time series and global field samples. *Environ. Microbiol.* **18**, 1403-1414 (2016).
7. A. E. Dekas *et al.*, Characterizing chemoautotrophy and heterotrophy in marine archaea and bacteria with single-cell multi-isotope NanoSIP. *Front. Microbiol.* **10**, 2682 (2019).
8. B. J. Callahan *et al.*, DADA2: High-resolution sample inference from Illumina amplicon data. *Nat. Methods* **13**, 581-583 (2016).
9. R.CoreTeam, R: A language and environment for statistical computing. Vienna, Austria. *Retrieved from* https://www.R-project.org/ (2016).
10. C. Lozupone, R. Knight, UniFrac: a new phylogenetic method for comparing microbial communities. *Appl. Environ. Microbiol.* **71**, 8228-8235 (2005).
11. J. Peplies, F. O. Glöckner, R. Amann, Optimization strategies for DNA microarray-based detection of bacteria with 16S rRNA-targeting oligonucleotide probes. *Appl. Environ. Microbiol.* **69**, 1397-1407 (2003).
12. W. Manz, R. Amann, W. Ludwig, M. Vancanneyt, K. H. Schleifer, Application of a suite of 16S rRNA-specific oligonucleotide probes designed to investigate bacteria of the phylum cytophaga-flavobacter-bacteroides in the natural environment. *Microbiology* **142**, 1097-1106 (1996).
13. R. Amann, J. Snaidr, M. Wagner, W. Ludwig, K. H. Schleifer, In situ visualization of high genetic diversity in a natural microbial community. *J Bacteriol* **178**, 3496-3500 (1996).
14. D. H. Parks *et al.*, A standardized bacterial taxonomy based on genome phylogeny substantially revises the tree of life. *Nat. Biotechnol.* **36**, 996-1004 (2018).
15. A. Pernthaler, J. Pernthaler, "Fluorescence *in situ* hybridization for the identification of environmental microbes" in Protocols for Nucleic Acid Analysis by Nonradioactive Probes, E. Hilario, J. Mackay, Eds. (Humana Press, Totowa, NJ, 2007), pp. 153-164.
16. C. A. Schneider, W. S. Rasband, K. W. Eliceiri, NIH Image to ImageJ: 25 years of image analysis. *Nat. Methods* **9**, 671-675 (2012).
17. R. I. Amann *et al.*, Combination of 16S rRNA-targeted oligonucleotide probes with flow cytometry for analyzing mixed microbial populations. *Appl Environ Microbiol* **56**, 1919-1925 (1990).
18. G. Wallner, R. Amann, W. Beisker, Optimizing fluorescent *in situ* hybridization with rRNA-targeted oligonucleotide probes for flow cytometric identification of microorganisms. *Cytometry* **14**, 136-143 (1993).
19. I. Klawonn *et al.*, Cell-specific nitrogen- and carbon-fixation of cyanobacteria in a temperate marine system (Baltic Sea). *Environ. Microbiol.* **18**, 4596-4609 (2016).
20. J. Lewin, Boron as a growth requirement for diatoms. *J. Phycol.* **2**, 160-163 (1966).
21. A. E. Dekas, V. J. Orphan, "Chapter Twelve - Identification of Diazotrophic Microorganisms in Marine Sediment via Fluorescence In Situ Hybridization Coupled to

Nanoscale Secondary Ion Mass Spectrometry (FISH-NanoSIMS)" in Methods in Enzymology, M. G. Klotz, Ed. (Academic Press, 2011), vol. 486, pp. 281-305.
22. H. Berthelot *et al.*, NanoSIMS single cell analyses reveal the contrasting nitrogen sources for small phytoplankton. *The ISME Journal* **13**, 651-662 (2019).
23. S. Gorka *et al.*, Rapid transfer of plant photosynthates to soil bacteria via ectomycorrhizal hyphae and its interaction with nitrogen availability. *Front. Microbiol.* **10**, 168 (2019).
24. M. T. Zumstein *et al.*, Biodegradation of synthetic polymers in soils: Tracking carbon into CO_2 and microbial biomass. *Sci. Adv.* **4**, eaas9024 (2018).
25. J. Pett-Ridge, P. K. Weber, NanoSIP: NanoSIMS applications for microbial biology. *Methods in molecular biology (Clifton, N.J.)* **881**, 375-408 (2012).
26. J. Pett-Ridge, P. K. Weber, NanoSIP: NanoSIMS applications for microbial biology. (in press).
27. N. Musat *et al.*, The effect of FISH and CARD-FISH on the isotopic composition of ^{13}C- and ^{15}N-labeled *Pseudomonas putida* cells measured by nanoSIMS. *Syst. Appl. Microbiol.* **37**, 267-276 (2014).
28. N. R. Meyer, J. L. Fortney, A. E. Dekas, NanoSIMS sample preparation decreases isotope enrichment: magnitude, variability and implications for single-cell rates of microbial activity. *Environ. Microbiol.* **23**, 81-89 (2021).
29. D. Woebken *et al.*, Revisiting N_2 fixation in Guerrero Negro intertidal microbial mats with a functional single-cell approach. *The ISME Journal* **9**, 485-496 (2015).
30. H. Stryhanyuk *et al.*, Calculation of single cell assimilation rates from SIP-nanoSIMS-derived isotope ratios: A comprehensive approach. *Front. Microbiol.* **9**, 2342 (2018).
31. R. R. L. Guillard, "Division rates" in Handbook of phycological methods: Culture methods and growth measurement, J. Stein, Ed. (Cambridge University Press, Cambridge UK, 1973), chap. 19, pp. 289-311.
32. A. S. Gsell *et al.*, Spatiotemporal variation in the distribution of chytrid parasites in diatom host populations. *Freshwat. Biol.* **58**, 523-537 (2013).
33. M. Kagami, E. Von Elert, B. W. Ibelings, A. De Bruin, E. Van Donk, The parasitic chytrid, *Zygorhizidium*, facilitates the growth of the cladoceran zooplankter, *Daphnia*, in cultures of the inedible alga, *Asterionella*. *Proc. R. Soc. B Biol. Sci.* **274**, 1561-1566 (2007).
34. K. Bruning, Infection of the diatom *Asterionella* by a chytrid. I. Effects of light on reproduction and infectivity of the parasite. *J. Plankton Res.* **13**, 103-117 (1991).
35. N. Kroer, Relationships between biovolume and carbon and nitrogen content of bacterioplankton. *FEMS Microbiol. Ecol.* **13**, 217-223 (1994).
